# Optimising Fluvoxamine Maternal/Fetal Exposure during Gestation: A Pharmacokinetic Virtual Clinical Trials Study

**DOI:** 10.3390/metabo12121281

**Published:** 2022-12-16

**Authors:** Khairulanwar Burhanuddin, Raj Badhan

**Affiliations:** School of Pharmacy, College of Health and Life Science, Aston University, Birmingham B4 7ET, UK

**Keywords:** depression, pregnancy, fetal, fluvoxamine, pharmacokinetics

## Abstract

Fluvoxamine plasma concentrations have been shown to decrease throughout pregnancy. CYP2D6 polymorphisms significantly influence these changes. However, knowledge of an optimum dose adjustment according to the CYP2D6 phenotype is still limited. This study implemented a physiologically based pharmacokinetic modelling approach to assess the gestational changes in fluvoxamine maternal and umbilical cord concentrations. The optimal dosing strategies during pregnancy were simulated, and the impact of CYP2D6 phenotypes on fluvoxamine maternal and fetal concentrations was considered. A significant decrease in fluvoxamine maternal plasma concentrations was noted during gestation. As for the fetal concentration, a substantial increase was noted for the poor metabolisers (PM), with a constant level in the ultrarapid (UM) and extensive (EM) metabolisers commencing from gestation week 20 to term. The optimum dosing regimen suggested for UM and EM reached a maximum dose of 300 mg daily at gestational weeks (GW) 15 and 35, respectively. In contrast, a stable dose of 100 mg daily throughout gestation for the PM is sufficient to maintain the fluvoxamine plasma concentration within the therapeutic window (60–230 ng/mL). Dose adjustment during pregnancy is required for fluvoxamine, particularly for UM and EM, to maintain efficacy throughout the gestational period.

## 1. Introduction

The rates of pregnant women diagnosed with depression have been reported as high as 25%, with a higher prevalence in the second and third trimesters [1,2,3]. Proper treatment is vital because poor management may lead to a myriad of complications for the mother and the foetus, such as malnutrition due to poor diet, preterm deliveries, foetal growth retardation, and miscarriages [4]. Thus, ensuring the optimisation of doses through gestation is essential; accordingly, plasma concentration levels are used as a guide in this respect [5]. In terms of the treatment selection, the use of selective serotonin reuptake inhibitors (SSRI) such as fluoxetine, fluvoxamine, paroxetine, sertraline, citalopram, and escitalopram usage has increased over the years from 1.5% in 1996 to between 3–6% in the last decade [6,7].

Fluvoxamine is used for the treatment of several conditions, such as major depression, obsessive-compulsive disorder (OCD), and social anxiety disorder. In addition, fluvoxamine has also been used in an off-labelled manner for various indications, such as post-traumatic stress disorder (PTSD), panic disorder, binge-eating disorder, and others [8,9,10,11]. Before the Pregnancy and Lactation Labeling Final Rule (PLLR) was implemented by the United States Food and Drug Administration (USFDA) in 2015, fluvoxamine was in category C of pregnancy risk based on the adverse effects noted in the foetus in a non-clinical study on pregnant rats, but no adequate information in humans was presented in order to draw conclusions from the findings [12,13]. This has been updated to highlight that no clear associated risk of significant congenital disability or miscarriage was linked with fluvoxamine usage based on several human observational studies [14]. In the context of the post-natal period, SSRIs have been reported to lead to Post Natal Adaptation Syndrome (PNAS), in which case they cross the placenta, and this traversal may result in increased concentrations in the developing foetus, thus impacting fetal respiratory, cardiovascular, and neurological development [15,16,17]. Unfortunately, information on fluvoxamine’s efficacy and plasma concentrations in the pregnant population is lacking, particularly with respect to a large-scale and well-controlled trial, which may be due to the ethical and safety concerns surrounding recruiting pregnant women as subjects. However, despite this lack of information, a study by Westin et al. [18] highlighted that fluvoxamine plasma concentrations significantly drop in the third trimester, possibly leading to ineffective treatment. However, further research is needed due to the small number of data. In addition, the impact of pregnancy on fluvoxamine plasma concentration levels suggests the need to explore the dosing regimens in the pregnant population.

Cytochrome P450 2D6 (CYP2D6) is a highly polymorphic drug-metabolism enzyme and is the primary hepatic enzyme responsible for fluvoxamine metabolism, with fluvoxamine acid being the major metabolite that is inactive and excreted through urine [19]. In this respect, a physiologically based pharmacokinetic (PBPK) simulation showed that dose increments are required for paroxetine, an antidepressant metabolised primarily by the same hepatic enzyme, in order to maintain the plasma concentration within the therapeutic window during gestation [20]. This result relates to an analysis of therapeutic drug-monitoring (TDM) services by Westin et al. [18], which showed that the fluvoxamine dose needs to be doubled to maintain the same plasma concentration as the prenatal period based on a linear mixed model analysis. However, the model was developed without considering the physiological changes that occurred throughout pregnancy and different CYP2D6 phenotypes.

The advancement of PBPK modelling with respect to simulating virtual clinical trials has provided a platform for addressing the scarcity of pharmacokinetic data, particularly in special populations such as pregnant women [20,21,22,23,24,25,26]. The physiological changes that occur during pregnancy are complex and include changes in cardiac output, plasma volume, body fat, protein binding, hepatic enzyme processes, and the glomerular filtration rate, which can impact drug distribution and excretion and may necessitate dosing adjustment to maintain a drug’s effectiveness [27,28,29,30,31]. The application of PBPK and virtual clinical trials in guiding the dose selection for the pregnant population has been applied for at least 46 compounds, of which 33 compounds showed that dose adjustment might be needed, particularly for the drugs that were metabolised extensively by hepatic enzymes [22].

Due to a paucity of fluvoxamine-related pharmacokinetic data on the pregnant populations, we have, for the first time, applied the concept of PBPK and virtual clinical trials in assessing the influence of pregnancy on both maternal and foetal fluvoxamine plasma concentrations. Furthermore, we have identified a dosing regimen for pregnant women considering the CYP2D6 phenotype status to maintain the plasma concentration within the therapeutic window during the perinatal period. This study aimed to utilise the concept of mechanistic, pharmacokinetic modelling and virtual clinical trials to: (1) evaluate the impact of gestational changes on fluvoxamine maternal and foetal concentration levels; (2) elucidate the influence of CYP2D6 polymorphism on maternal and foetal concentrations; and (3) determine the optimal dosing adjustment strategy considering the CYP2D6 phenotype status throughout gestation.

## 2. Materials and Methods

This study used the PBPK modelling tool, Simcyp^®^ Version 20 (Simcyp Ltd., Certara, Sheffield, UK), to develop and conduct virtual clinical trials on both healthy and pregnant subjects.

The Simcyp Simulator implements a minimal or full-body PBPK model. The former is a “lumped” 4-compartment model and considers systemic, portal vein, and liver concentrations with the addition of a “single adjusting compartment” representing a lump of all tissues except for the liver and portal vein. The full PBPK model is a generic, whole-body, 14-compartment model with the ability to incorporate additional compartments, such as a foeto-placental unit during pregnancy.

**Figure 1:**
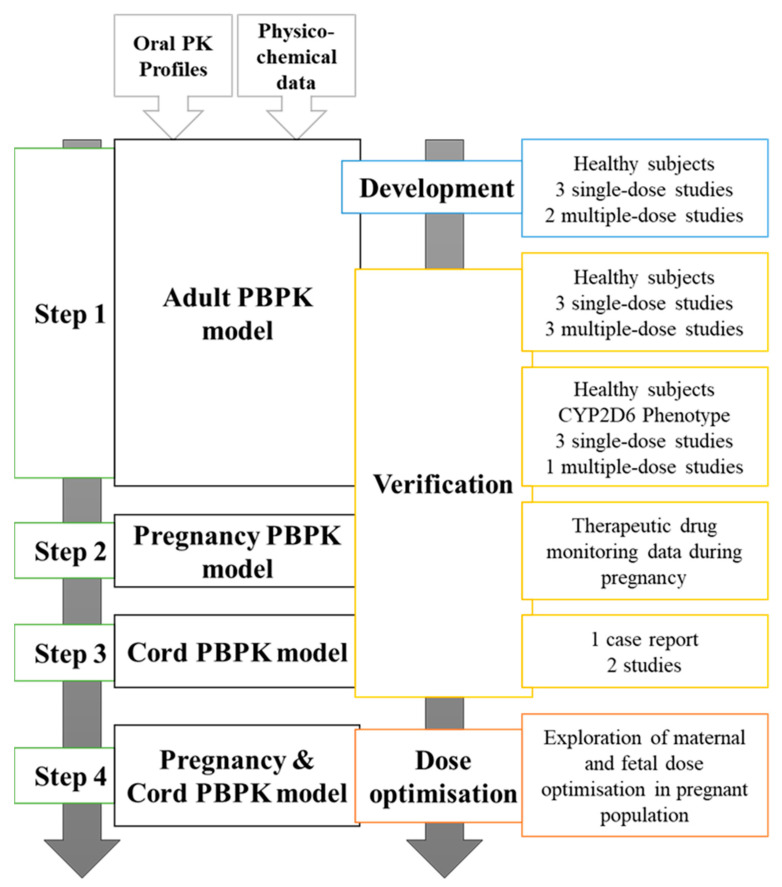
A 4-step workflow for fluvoxamine gestational model’s development.

We implemented a 4-step workflow to develop, validate, and simulate studies with fluvoxamine (Figure 1).

### 2.1. Step 1: Development and Verification of Fluvoxamine Model in a Healthy Population

We used the “healthy volunteer” (HV) population group available in Simcyp^®^ for simulation as a baseline population for non-pregnant females.

We employed the fluvoxamine compound file developed by Simcyp^®^, which is available in the simulator, with modifications made to a few parameters. First, the distribution model was changed from a minimal-PBPK model to a full-body PBPK distribution model with an estimation of tissue partition coefficients (K_p_) to calculate the volume of distribution (V_ss_) using the Rogers and Rowland approach [32,33]. The calculated V_ss_ was in line with several published studies [34,35]. The changes made to the distribution model are necessary to ensure that the tissue physiological temporal changes were considered throughout gestation when implementing the data on the pregnant population. Further, adaptations were made to the absorption rate constant (k_a_), fraction of dose absorbed (fa), and blood-to-plasma ratio (B/P) [36,37], with the final compound parameters detailed in Table 1.

We applied plasma concentration data from 3 single-dose and 3 multiple-dose studies to establish the fluvoxamine model and confirm modifications to the fluvoxamine compound. Thereafter, validation was conducted with 3 single-dose and 3 multiple-dose studies. In addition, we further validated the model using CYP2D6 extensive metaboliser (EM) and poor metaboliser (PM) populations with plasma concentration data published from 3 single-dose studies and 1 multiple-dose study. All studies used to develop and validate the amended fluvoxamine model are detailed in Table 2.

Virtual clinical trials were run in Simcyp^®^ with a 10 × 10 study design. The subjects’ ages, male-to-female ratio, and dosage regimens were correlated with the study design used in the development and verification stages.

### 2.2. Step 2: Validation of Fluvoxamine PBPK Model in Pregnancy

After developing and verifying the fluvoxamine model in the HV population, the pregnant population model developed by Simcyp^®^ was used for simulation. The pregnant population incorporated in the Simcyp^®^ simulator includes the essential physiological changes that occur throughout the gestational period. The pregnant population established in Simcyp^®^ incorporates the physiological changes in tissue composition/blood volume, renal/liver function, and temporal changes in enzyme activities throughout the maternal period, particularly with respect to CYP2D6, which plays an essential role in fluvoxamine metabolism [22,52,53].

Specifically, a gestational age-dependant function is incorporated into Simcyp Pregnancy to reflect the increase in CYP2D6 enzyme abundance throughout gestation and is based on a study by Ryu et al. [54], with the function (1) expressed as:

CYP2D6 (fold change in activity) = 1 × (1 + 0.0163 × GW + 0.0009 × GW^2^) (1)

where GW represents gestation week. This function is then propagated within the model to alter baseline CYP2D6 expression (9.4 pmol/mg protein) [55].

In order to validate the fluvoxamine model in the pregnant population, we simulated fluvoxamine pharmacokinetics in pregnant populations throughout the entire gestational period, using a 10 trials x 10 patients design. A 100 mg daily oral dose was utilised, and pharmacokinetic data samples were collected on the last 24 h of every 5th GW. As for baseline, a similar study design was simulated with a healthy female population dosed with 100 mg of fluvoxamine daily. Then, we verified the simulated steady-state trough plasma concentrations with observed data from TDM services in Norway published by Westin et al. [18]. The data were collated from 3 pregnant women taking 100 mg of fluvoxamine per day, consisting of 3 serum drug concentrations at baseline and 5 serum drug concentrations during pregnancy. The data presented individually allowed for extraction and comparison with the fluvoxamine model simulated in the pregnant population.

After verifying the fluvoxamine-administration-during-pregnancy virtual trial simulation, we explored the fluvoxamine plasma concentration trend. We applied the therapeutic range for fluvoxamine recommended by Consensus Guidelines for Therapeutic Drug Monitoring in Neuropsychopharmacology: Update 2017 [5] as a guide to review the effective level of fluvoxamine plasma concentration during pregnancy as an antidepressant from the TDM perspective. The recommended range is between 60–230 ng/mL [5].

### 2.3. Step 3: Validation of Fluvoxamine Fetoplacental PBPK Model

In order to predict foetal exposure, we utilised the fetoplacental model within the Simcyp Pregnancy model. This incorporates an “additional” set of compartments which account for the foetal blood and foetal lumped body, with the description of transplacental clearance. Simcyp^®^ uses a permeability-limited model for the foetoplacental compartment in Simcyp^®^. The model described the compound flux between the maternal, placental, and foetal clearance values with respect to the maternal-placental Cotyledon clearance values (CL_PDM_ and CL_PDF_) (Figure S1).

Given the paucity of data on fluvoxamine’s transplacental permeability, we used an in vitro–in vivo extrapolation (IVIVE) method reported by Winiwarter et al. [38] that utilises hydrogen bond donors (HBD), polar surface area (PSA), and correction for placental villous surface area to yield both CL_PDM_ and CL_PDF_ (Table 1). The placental villous surface area was derived from a meta-analysis of reported values and calculated using Equation (2) as follows:

Placental villous surface area (m^2^) = (0.135 × GW) − (0.023 × GW^2^) + (0.0015 × GW^3^) − (0.00002 × GW^4^) (2)

We simulated the umbilical cord concentration in the full-term pregnant population through a design consisting of 10 trials × 10 subjects. Then, we validated the predicted umbilical cord concentration with 3 observed umbilical cord concentrations from 3 different studies [56,57,58].

### 2.4. Step 3: Influence of CYP2D6 Phenotype and Dose Adjustment during Gestation

Considering that CYP2D6 is the main CYP enzyme involved in fluvoxamine metabolism, we validated the fluvoxamine PBPK model in terms of UM, EM, and PM CYP2D6 in healthy subjects. In addition, the various CYP2D6 metabolisers in the pregnant population in Simcyp^®^ have been validated by Almurjan et al. [20] for the paroxetine compound. Thus, we predicted the fluvoxamine plasma concentration profile in UM, EM, and PM CYP2D6 populations to assess the impact of CYP2D6 phenotype on plasma concentrations throughout gestation. We simulated a 10 × 10 trial design throughout the entire gestational period, with pharmacokinetic data samples collected during the last 24 h of every 5th GW from a population of entirely UM, EM, or PM CYP2D6 phenotypes.

The predictions covered a range of fluvoxamine doses from 50 mg daily to a maximum of 300 mg daily, with increments of 25 mg daily and doses above 150 mg daily administered in 2 divided doses. 

We assessed the influence of the CYP2D6 phenotype on pregnant women and its transference to the foetus at the starting dose of 50 mg daily and the minimum and maximum maintenance doses of 100 mg and 300 mg daily, respectively. Regarding dose adjustment, we assessed the percentage of (maternal) subjects with a peak concentration above 230 ng/mL and trough concentration below 60 ng/mL for every 5 GWs and each phenotype for every dose starting from 50 mg daily up to the maximum dose of 300 mg daily.

### 2.5. Prediction Performance

All the pharmacokinetics predictions made in the simulations that fell within 2-fold (0.5–2-fold) of published data were considered to represent ‘optimal’ predictive performance unless otherwise stated [59,60,61]. In addition, we verified the simulations visually using the visual predictive-checking (VPC) strategy [62]. This strategy was used to view all the simulated concentration–time profiles in steps 1, 2, and 3 in the observed/published data. The simulations were considered acceptable when the published profile overlapped and fell within the 5th and 95th percentiles of the predicted median concentration–time profile.

### 2.6. Data and Statistical Analysis

The data used for development and validation were extracted using WebPlotDigitizer version 4.5 (https://apps.automeris.io/wpd/) (accessed on 10 September 2022). In step 1, we conducted statistical analysis using a nonparametric, unpaired Student’s t-test to compare the observed and predicted data. In steps 2 and 4, the nonparametric one-way ANOVA with a Dunnett’s multiple comparisons post hoc test was used to compare the 5-weekly-simulated plasma concentration with the baseline (0) for maternal prediction and GW 20 for umbilical cord simulation. For the comparison between UM, EM, and PM CYP2D6 phenotypes for every 5-weekly-simulated plasma concentration in maternal and umbilical cord concentration, we used the nonparametric one-way ANOVA with a Tukey’s multiple comparisons post hoc test. The significance test was performed with *p* < 0.05 for steps 1, 2, and 4. A statistical analysis was run using GraphPad Prism Version 8 for Windows (GraphPad Software, La Jolla, CA, USA).

## 3. Results

### 3.1. Step 1: Development and Validation of Fluvoxamine Model in a Healthy Population

The fluvoxamine model was adapted and validated using clinical studies, which included both single- and multiple-dose studies with various dosing regimens (Table 2). The predicted pharmacokinetic parameters, including C_max_, T_max_, AUC_0-t_, and AUC_inf_, were within 0.5 to 2-fold of the reported clinical data (Table 3). Moreover, the observed profiles agree with the simulated profile for single and multiple-dose studies based on the VPC, wherein the published profiles are within the 5th and 95th percentiles of the predicted plasma-concentration profile, thereby confirming the successful development and validation of the fluvoxamine model in the healthy population.

**Figure 2:**
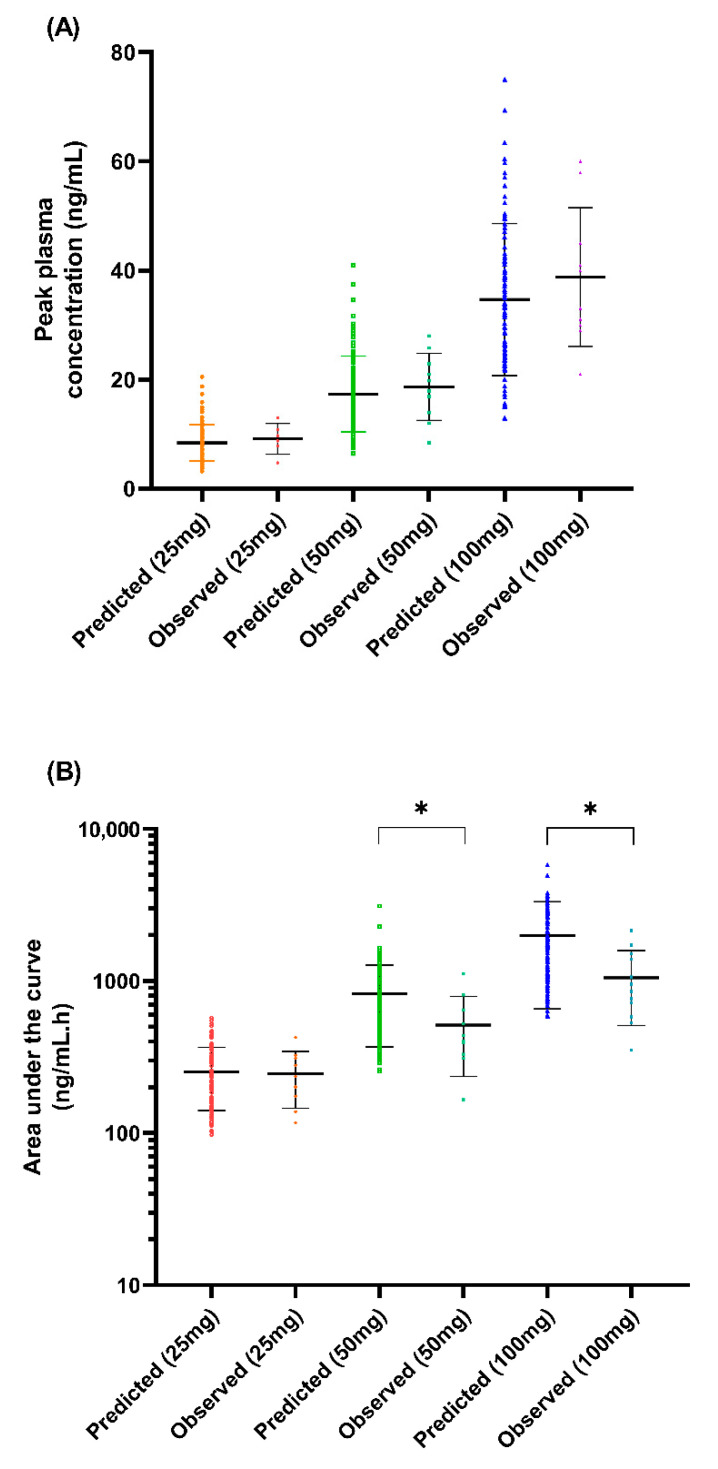
Comparison between simulated trial and observed data for (**A**) C_max_ and (**B**) AUC_inf_ from De Vries et al. [39]. Coloured data points arranged vertically represent the predicted and observed data for each dose; horizontal lines on the coloured data points represent the mean and standard deviation (SD). * *p* < 0.05.

**Figure 3:**
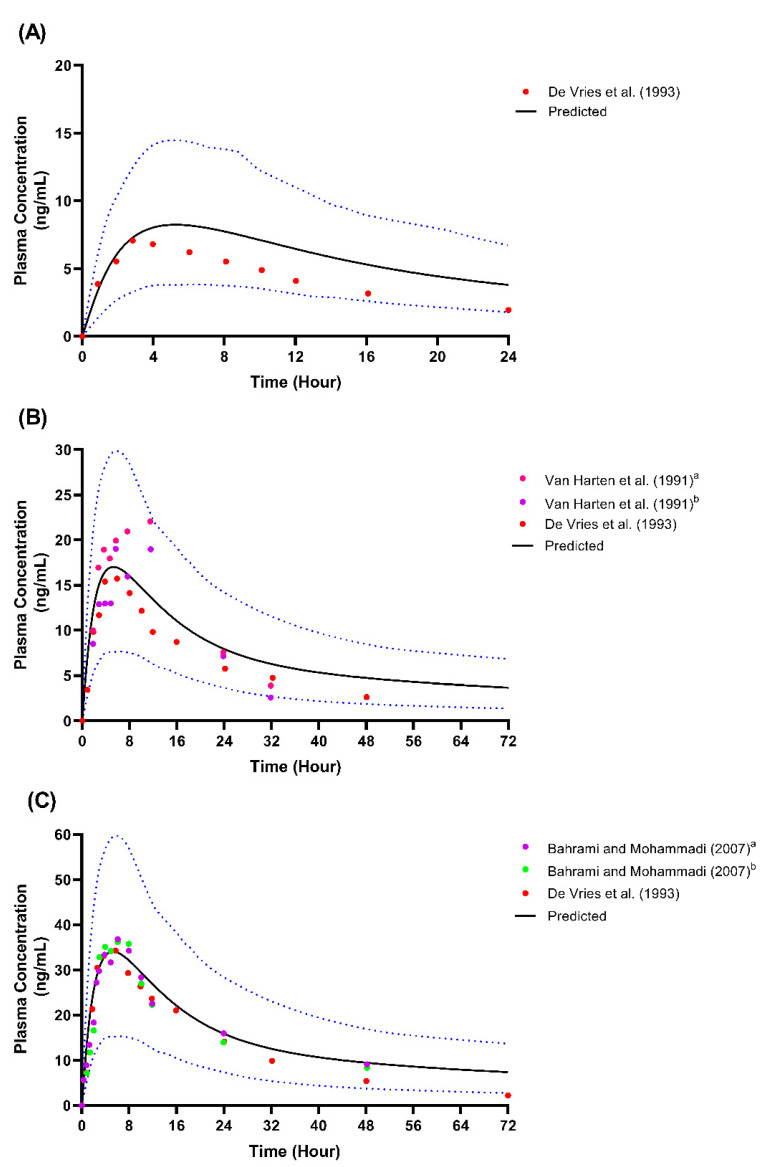
Single-dose studies simulated through model development. (**A**) Single-dose 25 mg; (**B**) Single-dose 50 mg; (**C**) Single-dose 100 mg. Solid lines represent the mean predicted concentration-time profile, with dotted lines representing the 5th and 95th percentile ranges. Solid circles represent observed clinical data from each study. Van Harten et al. (1991)^a^ represents the 50 mg fed study [40]; Van Harten et al. (1991)^b^ represents the 50 mg fast study [40]. Bahrami and Mohammadi (2007)^a^ represents the 100 mg test formulation study [41]; Bahrami and Mohammadi (2007)^b^ represents the 100 mg reference formulation study [41].

**Figure 4:**
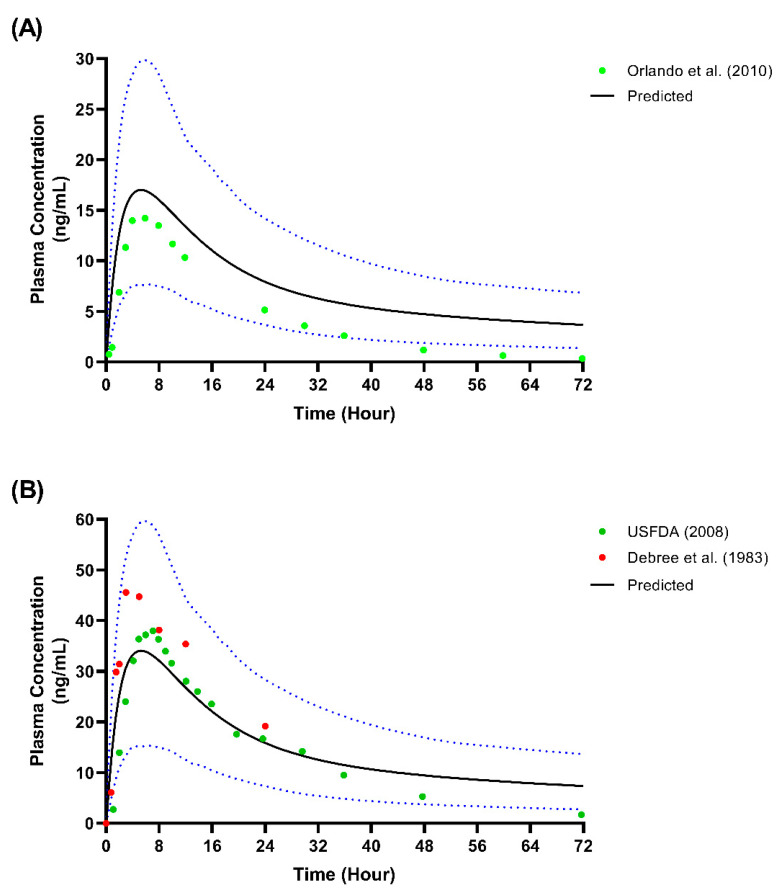
Single-dose studies simulated in model validation stage. (**A**) Single-dose 50 mg [44]; (**B**) Single-dose 100 mg [45,46]. Solid lines represent the mean predicted concentration-time profile, with dotted lines representing the 5th and 95th percentile ranges. Solid circles represent observed clinical data from each study.

We presented the simulated plasma concentration for all the single-dose studies used during the model’s development and validation in Figure 2, Figure 3 and Figure 4. For the comparison of the pharmacokinetic parameters in the single-dose studies, the AUC_inf_ was not within the limit determined from the model’s validation, particularly with respect to the study by Orlando et al. [44] and the USFDA [46]. A similar pattern was observed for the AUC_inf_ data when we compared the single-dose 50 mg and 100 mg trials conducted by De Vries et al. [39] with the spread of the individual data from the simulated profiles during the model’s development, as shown in Figure 2. However, the results showed no statistically significant difference (*p* > 0.05) for all three doses of C_max_ and the AUC_inf_ of the single-dose of 25 mg.

**Figure 5:**
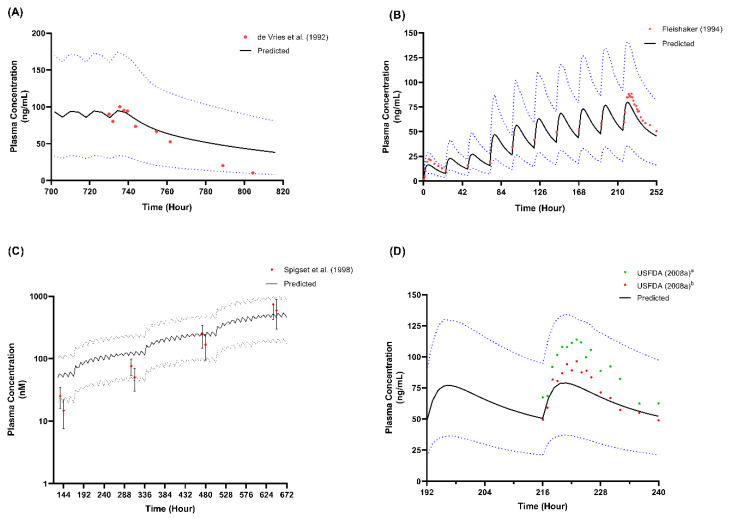
Multiple-dose studies simulated in model development and validation. (**A**) Multiple-dose 50 mg twice daily from day 4 to day 31 [42]; (**B**) multiple-dose 50 mg daily for 3 days followed by 100 mg daily for 7 days [43]; (**C**) multiple-dose 12.5 mg twice daily for week 1, 25 mg twice daily for week 2, 50 mg twice daily for week 3, and 100 mg twice daily for week 4 [47]; (**D**) multiple-dose 100 mg daily for 10 days [46]. Solid lines represent the mean predicted concentration-time profile, with dotted lines representing the 5th and 95th percentile ranges. Solid circles represent observed clinical data from each study, with error bars indicating SD. USFDA (2008)^a^ represents a bioavailability study with prototype D [46]; USFDA (2008)^b^ represents a bioavailability study with prototype C [46].

Regarding the multiple-dose study, only the C_max_ and AUC_inf_ for the study by Spigset et al. [47] were not within the 2-fold range, and this was when fluvoxamine was administered at the lowest dose at week 1 (12.5 mg twice daily for 7 days). The simulated plasma-concentration profiles and the published concentration data used during the development and verification of the multiple-dose studies are shown in Figure 5. 

**Figure 6:**
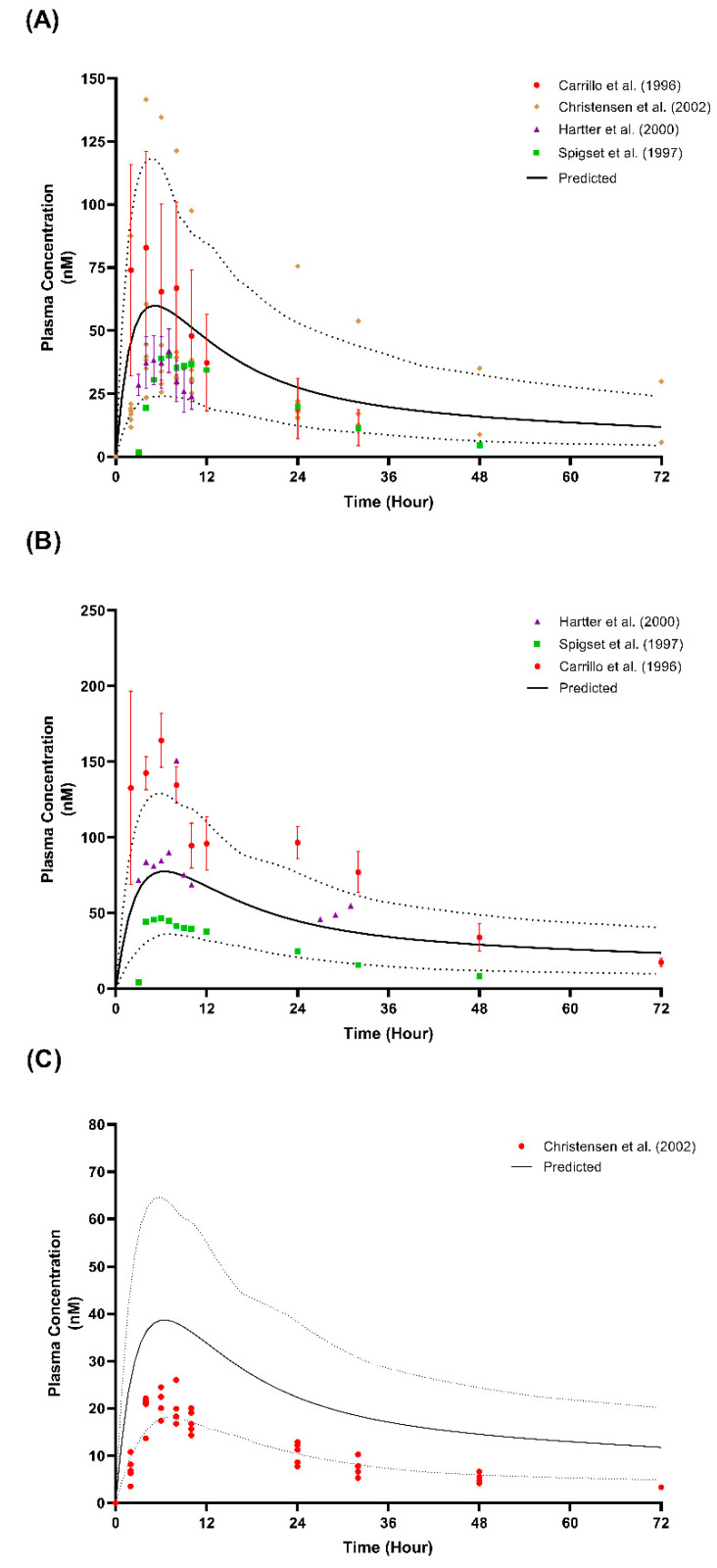
Simulated single-dose studies in model validation for CYP2D6 phenotype. (**A**) Single-dose 50 mg in EM CYP2D6 population [48,49,50,51]; (**B**) single-dose 50 mg in PM CYP2D6 population [48,49,50]; (**C**) single-dose 25 mg in PM CYP2D6 population [51]; solid lines represent the mean predicted concentration-time profile, with dotted lines representing the 5th and 95th percentile ranges. Solid circles represent observed clinical data from each study, with error bars indicating SD.

**Figure 7:**
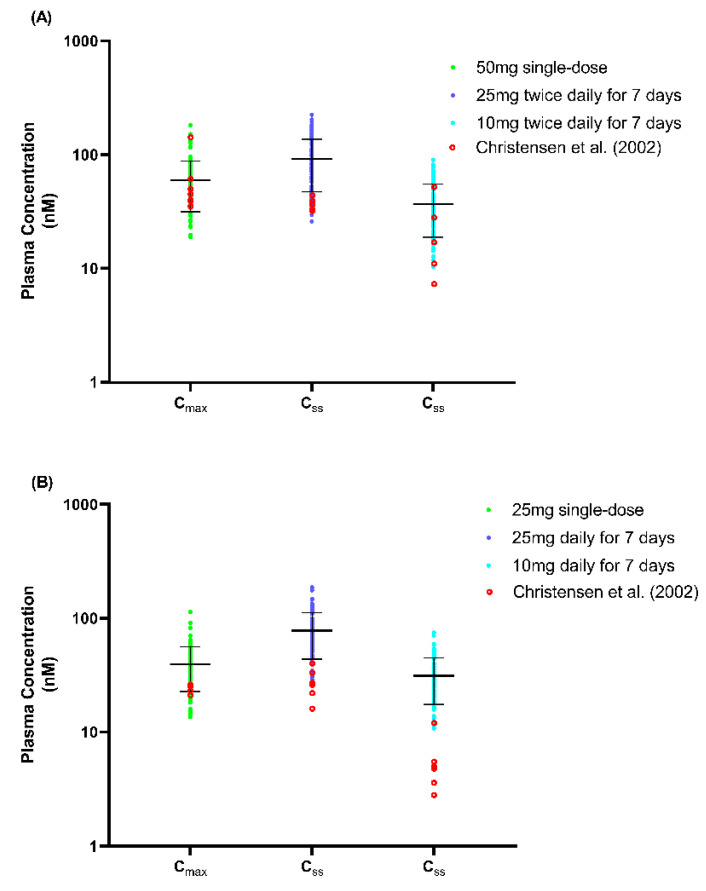
Predicted maximum concentration and steady-state concentration for single-dose and multiple-dose studies. (**A**) EM CYP2D6 phenotype population; (**B**) PM CYP2D6 phenotype population; solid circles arranged vertically represent the predicted values for each dose. Horizontal lines on the coloured data points represent the mean and SD. Red, open circles represent the observed individual data from Christensen et al. [51]. C_max_, maximum concentration for single-dose; C_ss_, average trough concentration at steady-state for Day 6 and Day 7.

The validation of the predicted values overlayed with the observed plasma concentrations for the graphs of the CYP2D6 EM and PM populations are presented in Figure 6 and Figure 7. Regarding the comparison of the pharmacokinetics parameters, a few parameters that were not within the 2-fold range were only seen in the single-dose 50 mg study by Spigest et al. [49] concerning the AUC_inf_ in both the EM and PM and the AUC_0-t_ for the PM, as well as the C_max_ for PM CYP2D6 for the single-dose 50 mg study by Carrilo et al. [48].

### 3.2. Step 2: Verification of Fluvoxamine Model in Pregnancy and the Impact of Pregnancy on Fluvoxamine Level

**Figure 8:**
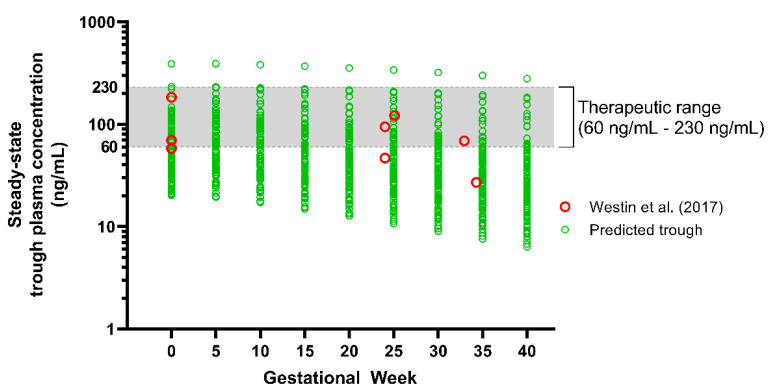
Predicted steady-state C_min_ fluvoxamine maternal concentration. Green, open circles represent the post-dose trough concentration sampled at 24 h post-dose and assembled every 5 GWs throughout the maternity period. Red, open circles represent reported plasma concentrations collected from 3 pregnant women from Westin et al. [18]. ‘0’ refers to the baseline predicted in the non-pregnant female population. The grey shaded region represents the fluvoxamine therapeutic window (TW).

In order to verify the applicability of the model throughout gestation, we validated the predicted fluvoxamine steady-state trough plasma concentrations (C_min_) following a daily 100 mg dose throughout pregnancy, with the reported TDM trough concentrations data throughout gestation reported by Westin et al. [18] (Figure 8). The model predictions were within the range reported by Westin et al. [18], with mean plasma concentrations showing a reducing trend from GW 10 towards term (Table 4).

When compared to the baseline, C_min_ and C_max_ started to decrease from GW 10 by −5.13% and −5.69%, and −48.46% and −49.37% in GW 40, respectively. Furthermore, we noticed that the decrease was statistically significant compared to the baseline commencing from GW 25 and 20 onwards for the C_min_ and C_max_, respectively. The trend showed that the mean of C_min_ falls below the therapeutic window at GW 25 onwards. The percentage of subjects with C_min_ below 60 ng/mL increased at the early stage of the 3rd trimester (GW 30) and up to 85% at GW 40. A similar trend was noted for C_max_. As for the mean, the C_min_ started to fall below the therapeutic concentration at GW 20 with 59.84 ± 51.76 ng/mL.

### 3.3. Step 3: Validation of Fluvoxamine Fetoplacental PBPK Model

**Figure 9:**
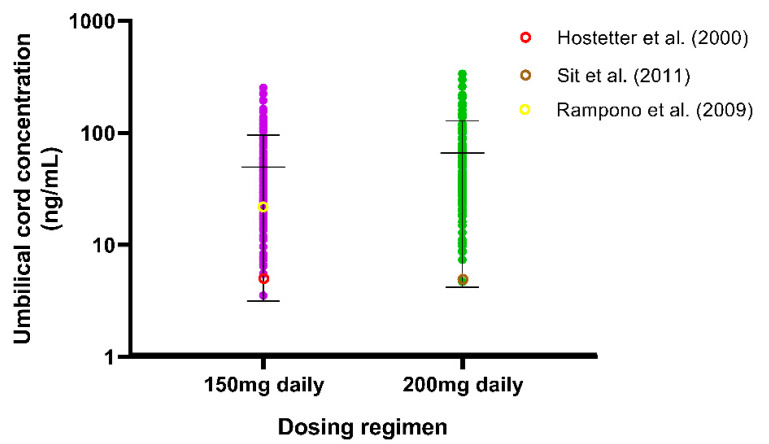
Simulated fluvoxamine foetal (umbilical cord) concentrations. Doses were administered to steady-state with sampling on the final 30-h period of GW 40. Solid circles represent individual predicted cord concentrations. Coloured open circles represent the observed umbilical cord concentrations from Hostetter et al. [56], Sit et al. [57] and Rampono et al. [58],. Horizontal lines on the coloured data points represent the mean and SD.

Since there is a higher risk of congenital disabilities for newborns of women treated with SSRIs, we developed and validated a fluvoxamine foetoplacental PBPK model to review the trend regarding the fluvoxamine levels in the umbilical cord. We validated the model only by the VPC with the reported values by Hostetter et al. [56], Sit et al. [57], and Rampono et al. [58]. Even though the individual observed values are sparse, the values fall within the range of the predicted cord concentrations (Figure 9).

### 3.4. Step 4: Impact of CYP2D6 Phenotype and Dose Adjustment during Gestation

**Figure 10:**
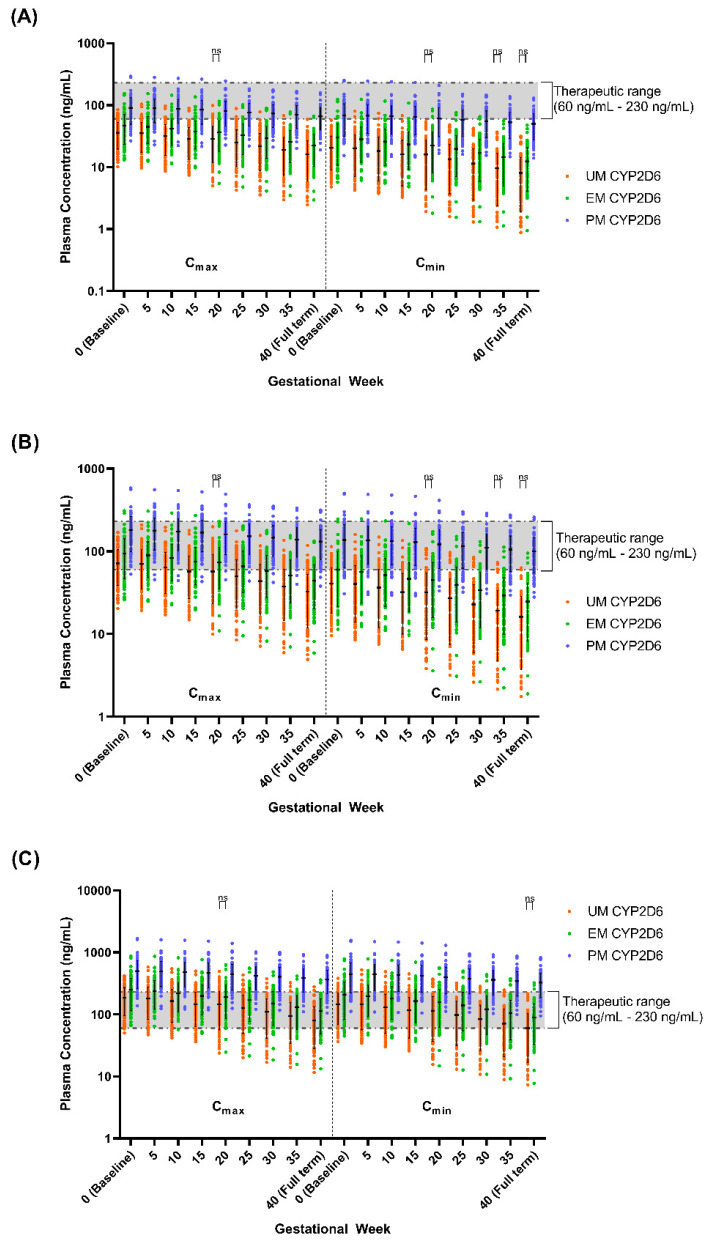
Simulated fluvoxamine maternal concentrations in CYP2D6 phenotype population. (**A**) 50 mg daily; (**B**) 100 mg daily; (**C**) 300 mg daily. Coloured solid circles represent individual, predicted maternal concentrations. C_max_, maximum concentration; C_min_, minimum concentration. Horizontal lines on the coloured solid circles represent mean and standard deviations. The shaded region represents the fluvoxamine TW. Comparison between each CYP2D6 phenotype for every 5 GWs showed statistically significant difference except between UM and EM at GW labelled as ‘ns’, *p * > 0.05.

**Figure 11:**
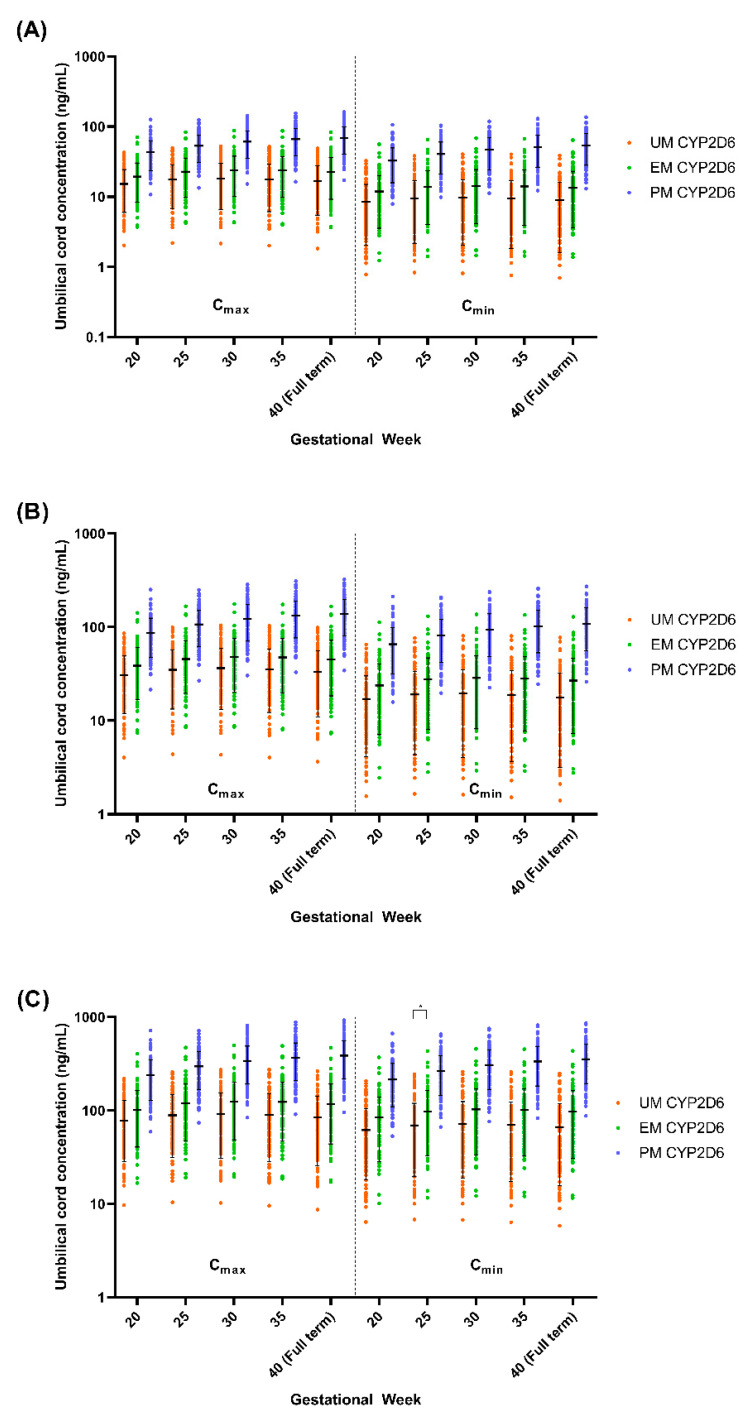
Simulated fluvoxamine umbilical cord concentrations in CYP2D6 phenotype population. (**A**) 50 mg daily; (**B**) 100 mg daily; (**C**) 300 mg daily. Coloured solid circles represent individual, predicted umbilical cord concentrations. C_max_, maximum concentration; C_min_, minimum concentration. Horizontal lines on the coloured solid circles represent mean and standard deviations. Comparing each CYP2D6 phenotype for every 5 GWs starting from GW 20 showed a statistically significant difference when compared with PM and a non-statistically significant difference between UM and EM at GW 25 for 300 mg daily—labelled as ‘*’; *p* < 0.05.

Given the several-fold increase in the C_max_, AUC, and t_½_ in PM CYP2D6 compared to the EM CYP2D6 [14], we explored the impact of the CYP2D6 phenotype on the fluvoxamine levels in the pregnant population. We compared the plasma concentration levels for both the mother (GW 0–40) and umbilical cord (GW 20–40) between the UM, EM, and PM CYP2D6 phenotypes (Figure 10 and Figure 11) and the changes as compared to the baseline (0) for the mother (Table 5), while the percentage changes regarding the umbilical cord concentration from GW 20 are reported in Table 6. 

We noticed a statistically significant difference between the UM and EM with respect to the PM CYP2D6 phenotype population for each 5th GW across all three doses (Figure 10). A similar pattern was seen between the UM and EM CYP2D6 populations with few exceptions, particularly in GW 20 regarding the C_max_ and C_min_, when the foetoplacental PBPK model was initiated (Figure 9). However, the significant difference is minimal as compared to the PM. As for the cord concentration, the difference was significant with the PM but not between the UM and EM CYP2D6 populations across all five GWs (Figure 10). Since statistically significant differences were seen between the UM and EM CYP2D6 populations, we explored the dosing regimens for each of the CYP2D6 phenotype populations. 

Looking at the concentration trend (Table 5), we identified the same pattern for both the UM and EM populations, with the concentration significantly decreased across all three doses starting from GW 15 in both the peak and trough. Whereas for the PM population, the concentration began to drop significantly from GW 25 for the peak and GW 30 for the trough, except at the 300 mg daily dose, where the decrease started to be statistically significant at GW 25. These patterns concur with the concentration trend in the general pregnant population reported in Step 2. 

Moreover, for the 50 mg daily dose at GW 40, both the trough and peak levels demonstrated 60.40% and 54.77% decreases for the UM population and 59.01% and 52.76% decreases for the EM population when compared to the baseline. Whereas for the PM population, we saw 27.19% and 27.27% decreases for the trough and peak, respectively. This pattern is comparable across the 100 mg and 300 mg daily doses (Table 5). 

Regarding the foetal cord level, both the trough and peak concentrations increased at full term compared to GW 20, and this transpired at all three-dose levels and CYP2D6 phenotype populations (Table 6). The PM CYP2D6 population demonstrated a significant increase across all GWs at the 50 mg daily dose (trough, 23.95% at GW 25 vs. 64.58% at full term; peak, 23.98% at GW 25 vs 60.88% at full term), 100 mg daily dose (trough, 23.95% at GW 25 vs. 64.58% at full term; peak, 23.98% at GW 25 vs. 60.87% at full term), and 300 mg daily dose (trough, 24.09% at GW 25 vs 64.82% at full term; peak, 23.92% at GW 25 vs. 61.64% at full term). Unlike the PM population, UM and EM have the same trend, in which the cord level increases until GW 30 and decreases back until the full term, with a significant difference only seen between GW 30 and GW 20 for the peak of the EM CYP2D6 population in the 50 mg daily and 100 mg daily doses (Table 6). 

The percentage of subjects where the trough level falls below 60 ng/mL is more than 50% for both the UM and EM populations at doses of 50 mg daily and 100 mg daily. In contrast, with respect to the 300 mg daily dose, for the UM population, this trend started from GW 35 when the C_min_ fell below 60 ng/mL for more than 50% of the subjects and did not reach 40% of the subjects for the EM population. As for the PM population, the percentage of subjects for whom the peak level rose above 230 ng/mL is more than 90% for the 300 mg daily dose; for more than 40% of the subjects, the peak level falls below 60 ng/mL (Table 5). 

Since the percentage of subjects where the peak and trough levels fall outside the therapeutic windows varies between the different phenotypes of the CYP2D6 populations, we used the threshold of 20% outside of the therapeutic windows to determine the suitable dose for the pregnant population according to their phenotype (Figures S2–S7) [20,23,24].

For the UM CYP2D6 population, a fluvoxamine dose of 250 mg or 275 mg daily in the first trimester, followed by a maximum dose of 300 mg daily until the full term, is suggested to be optimum, as it corresponds to point at which the maternal concentrations are within the therapeutic windows for most of the subjects (Table 7). Nevertheless, for the maximum dose of 300 mg daily, the percentage of subjects with a C_min_ below 60 ng/mL is between 21% in GW 15 to 65% in the full term, but none of the peak concentrations are above 230 ng/mL (Table 7, Figures S2 and S3). 

For EM, a fluvoxamine dose of 175 mg daily is suitable up to GW 10, a 200 mg daily dose is ideal up to GW 15, and a 225 mg daily dose is advisable between GW 5 to 20, which covers the first trimester and the early second trimester. A 250 mg daily dose can be used for GW 10 to 25, while a 275 mg daily dose is effective between GW 15 to 30, covering the second trimester. As for GW 30 to full term, a maximum dose of 300 mg daily is considered the most effective since it had the most subjects where the C_min_ and C_max_ fell within the therapeutic range (Table 7 and Table 8; Figures S4 and S5).

With regard to PM, this approach revealed that a doe of 75 mg daily is suitable for GW 10, while a 100 mg daily dose is effective throughout pregnancy. It is also possible to increase the dose to 125 mg daily at GW 35 until labour, as the percentage of subjects for which the trough and peak fall within the therapeutic window is 8% and 9% for the trough and 19% and 16% for the peak for GW 35 and 40, respectively (Table 7, Figures S6 and S7).

**Figure 12:**
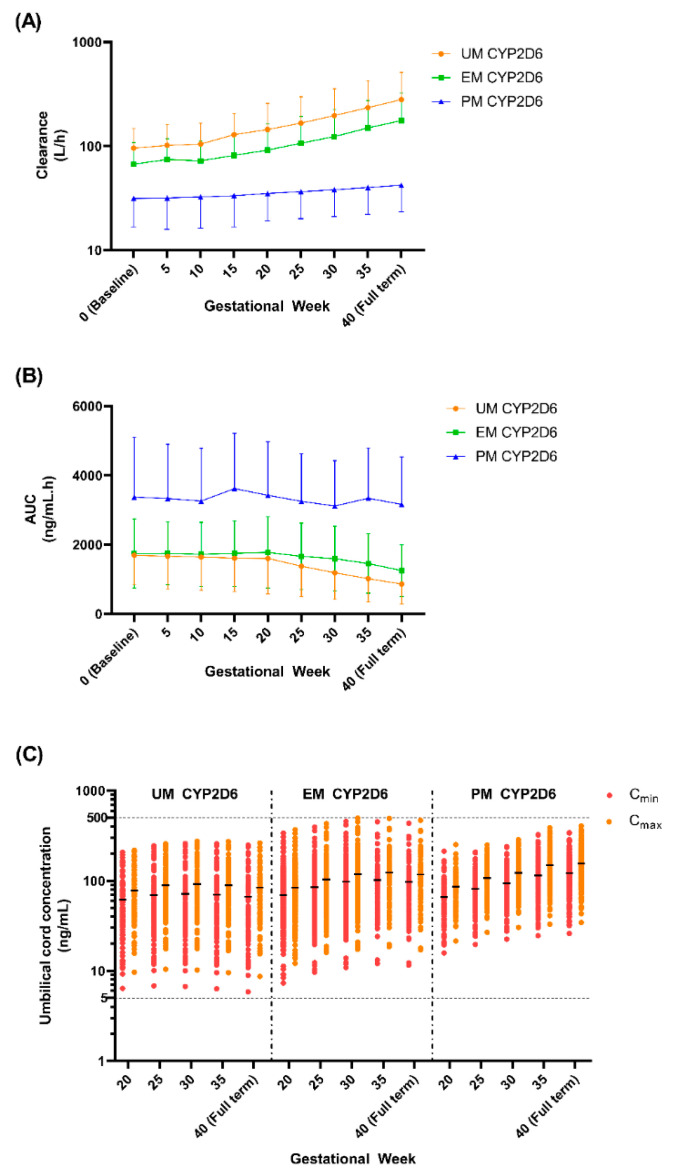
Predicted clearance, area-under-the-curve, and cord concentration based on the recommended doses. (**A**) Clearance; (**B**) area-under-the-curve; (**C**) umbilical cord concentration. Top and bottom horizontal lines in (**A**,**B**) represent standard deviations. Coloured, closed circles in (**C**) are the predicted individual cord concentrations. Horizontal lines on the coloured, solid circles in (**C**) represent the mean. Dashed horizontal lines in (**C**) represent the range of simulated cord concentrations throughout gestational periods and all three CYP2D6 phenotypes (5 ng/mL to 500 ng/mL).

We noticed a gradual increase in the clearance from GW 5 to GW 30 for both the UM and EM CYP2D6 populations, while the clearance is constant throughout pregnancy for the PM population (Figure 12 and Table 8). Likewise, the AUC remained steady throughout pregnancy for the PM population, while the AUC slightly decreased starting from GW 25 to full term for the UM and EM populations (Figure 12 and Table 8). This trend is expected since the suggested doses are higher as the pregnancy is near the full term for both UM and EM, but for PM, the recommended dose is maintained throughout the gestational period. 

Based on the recommended dose, the range of the expected fluvoxamine concentration that crosses the placenta is between 5.84 ng/mL to 496.10 ng/mL across the gestational period and the CYP2D6 phenotype (Figure 12). We have seen a similar trend in both the UM and EM populations, wherein the foetal concentration increased until GW 30 and then became stagnant until labour. Whereas the cord concentrations steadily increase for the PM population until full term (Table 8). 

## 4. Discussion

Several observational studies have demonstrated that SSRIs, including fluvoxamine, are safe to use during pregnancy, even given the possible risk of persistent pulmonary hypertension (PPHN), for which the benefit of controlling major depression may outweigh the risk depending on the patient’s situation [63,64,65,66,67,68,69]. However, the efficacy and impact of antidepressants in the pregnant population, particularly for fluvoxamine, are still lacking because no controlled trials have been conducted on the pregnant population.

Therapeutic drug monitoring (TDM) is one approach that can offer dose adjustment throughout gestation; however, this is often not considered viable or necessary for many drugs. However, the use of robust and validated mechanistic pharmacokinetic modelling allows for an assessment of any changes that occur in a drug’s PK properties during the gestational period, one which considers the physiological changes during pregnancy and offers a pragmatic solution to the following question: “what is the correct dose during gestation?” [5,20,22,23,24,25,26,70]. Although the concept has been utilised for other compounds, this is the first time it has been used to develop a fluvoxamine PBPK pregnancy model to support material dosing and foetal exposure.

### 4.1. Step 1: Validation of Fluvoxamine Model in Healthy Subjects

#### 4.1.1. PBPK Model Parameters

The modification of a minimal-PBPK model to a full-body PBPK distribution model was essential to ensure that the physiological changes that occurred throughout the gestational period were considered for the PBPK pregnancy model [20,23,24,25,26]. Furthermore, the estimation of K_p_ developed to predict V_ss_ using the Rogers and Rowland approach [32,33] resulted in a V_ss_ within two-fold of the published V_d_ [34,35]. In addition, the k_a,_ fa, and B/P were amended based on published data and the Simcyp^®^ prediction [36,37]. Finally, the modifications were guided by three single-dose and two multiple-dose studies, which were further validated through another three single-dose and three multiple-dose studies incorporating healthy subjects and four single-dose studies and one multiple-dose study in CYP2D6 phenotype populations.

#### 4.1.2. Validation in Healthy Subjects and CYP2D6 Phenotype Populations

The predicted PK parameters were within two-fold of the published PK studies, except for the AUC_inf_ (Table 3). Furthermore, a similar pattern was seen for the individual AUC_inf_ comparison between the observed data by De Vries et al. [39] and the prediction, which showed a statistically significant difference for the 50 mg and 100 mg formulations but not for the 25 mg formulation, the overlayed PK profile, and other PK parameters including the AUC_0-t_. The AUC_inf_ is not commonly used for comparison among PK parameters, particularly in the regulatory setting, due to its reliability, specifically when the percentage differences between AUC_inf_ and AUC_0-t_ are more than 20%, as is the case here where the difference was not reported for the observed data, and the difference for the prediction is more than 20% [71]. Furthermore, the total number of sampling points used is crucial for an accurate estimation of AUC_inf_; in this situation, the number of samples used was notably different between the observed and predicted values (3 to 15 samples vs. > 100 samples), possibly overestimating the value of one over another [72].

The imperfect prediction of the lowest dose (12.5 mg twice daily) compared to that observed by Spigset et al.’s [47] study is compensated by a reliable prediction at the other three higher doses (25 mg twice daily, 50 mg twice daily, and 100 mg twice daily) (Table 3 and Figure 5). This result may be due to the dose being lower than the minimum daily dose recommended for adults, which is 50 mg administered once daily [14]. The prediction for the PM CYP2D6 population is not ideal when weighed individually with each published study. However, an assessment of the plasma concentration profile showed that the simulated profile matched all three published studies because of the wide variation between the studies (Figure 6). A similar phenomenon can be seen for the prediction of the multiple-dose study at the lowest dose (25 mg and 10 mg daily) when compared to the data observed by Christensen et al. [51], particularly for the PM population. The observed data fall in the lower range of the predicted data, which agrees with the prediction made by Britz et al. [73] for the fluvoxamine model (Figure 7).

Broader acceptance criteria, as discussed by Abduljalil et al. [74], may be considered, specifically for the PM population, since the comparisons were made between small subject samples from published works with 100 virtual patients for each simulated study and as the observed trials showed a wide variation of results. Nevertheless, the VPC strategy showed that the simulated PK profiles fall within the 5th and 95th percentiles of all the 14 studies. Therefore, these results validate the fluvoxamine PBPK model in the healthy population.

### 4.2. Step 2: Verification of Fluvoxamine Pregnancy Model and the Impact of Pregnancy on Fluvoxamine Concentration

#### 4.2.1. Verification of Fluvoxamine Pregnancy Model

Based on the literature review, the study by Westin et al. [18] is the only publication (to date) containing data on the fluvoxamine plasma concentration throughout the 40-week gestational period. Thus, these were the only data used to validate the fluvoxamine PBPK pregnancy model. Using the VPC strategy, the predicted fluvoxamine plasma concentrations followed the pattern of the published data throughout the gestational period. Furthermore, the results showed that the difference compared to the baseline was significant from GW 20 and GW 25 for C_max_ and C_min_, respectively, which is consistent with the published data reported by Westin et al. [18].

#### 4.2.2. The Impact of Pregnancy on Fluvoxamine Concentration

The simulation demonstrated that out of 100 pregnancies, the C_min_ for more than 50% of pregnant women falls below the minimum effective concentration of 60 ng/mL recommended by Hiemke et al. [5] for the treatment of major depression. The trend showed that the number significantly increased, particularly after GW 25 in both C_min_ and C_max_, suggesting the need for fluvoxamine dose adjustment to maintain the same efficacy as pre-partum.

The possible main factor influencing the fluvoxamine concentration during the gestational period is hepatic enzyme metabolism, specifically, CYP2D6. This is because fluvoxamine is extensively metabolised in the liver, predominantly by CYP2D6, with minimal influence by CYP1A2 [14,19,47,75]. Thus, the reduction in fluvoxamine plasma concentration concurs with increasing CYP2D6 activities throughout pregnancy by 25.6% ± 58.3% at GW 14–18 to 47.8% ± 24.7% at GW 36–40 compared to the postpartum period [76]. The same trend has been reported by Wadelius et al. [77], but the study only performed the phenotyping at GW 36 instead of at every trimester. Furthermore, the increasing trend of the CYP2D6 enzyme’s activities throughout pregnancy has also been incorporated in Simcyp^®^ and validated based on pregnancy PBPK modelling for several compounds, namely, metoprolol and paroxetine, which are reflected in this study as well [20,70].

On the other hand, the decreasing trend in the fluvoxamine plasma concentration throughout the gestational period further supports the findings of several publications that show that the contribution of the CYP1A2 enzyme to fluvoxamine metabolism is not significant compared to the CYP2D6 enzyme [19,47,48,78]. The explanation behind this is that the opposite trend between CYP2D6 and CYP1A2 activities throughout pregnancy is supposed to cancel out the impact on drug plasma concentration during pregnancy if the fractional metabolism of a drug is equal between the two enzymes, which has not been seen in this study and the study by Westin et al. [18,52,70,76,79].

Since fluvoxamine is a lipophilic drug, other physiological changes may contribute to reducing the fluvoxamine levels, such as the expansion of intravascular and extravascular volume as well as the increase in body fat throughout the gestational period [52,80]. In contrast, although fluvoxamine is a basic drug, the influence of changes in gastric pH and gastrointestinal motility on fluvoxamine absorption may be minimal compared to hepatic metabolism since fluvoxamine is highly absorbed from the gastrointestinal tract with approximately 50% bioavailability [14].

Renal changes may have minimal influence since fluvoxamine is primarily eliminated through hepatic biotransformation with no known active metabolites, and a negligible amount of fluvoxamine is excreted unchanged in the urine [19]. Moreover, studies have shown that dose adjustment is needed for hepatic but not renal impairment patients [81,82,83,84].

### 4.3. Step 3: Validation of Fluvoxamine Fetoplacental PBPK Model

The sparse data obtained in this study were expected since another fluvoxamine foetoplacental model developed by Matsuoka et al. [85] was validated with data solely taken from Hostetter et al. [56]. The limited information on when the samples were taken after the last dose provides a challenge in simulating optimal timing in order to offer a fair comparison. Nevertheless, we validated the fluvoxamine foetoplacental model based on three published studies which showed that all the observed data fit within the standard deviation of the predicted concentrations. In addition, our predicted concentrations are comparable to those made by Matsuoka et al. [85], particularly for the 150 mg daily doses. The fluvoxamine foetoplacental model was developed without including any specific active transport mechanism other than passive diffusion, which is similar to Matsuoka et al.’s [85] model. Thus, the CYP2D6 activity in the mother is the main factor influencing the fluvoxamine level in the foetus.

### 4.4. Step 4: Impact of CYP2D6 Phenotype and Dose Adjustment during Gestation

#### 4.4.1. Impact of CYP2D6 Phenotype in the Pregnant Population

A notable difference was seen between the EM and PM CYP2D6 populations regarding the validation of the healthy subject models and the intrinsic hepatic clearance data from the verification stage in the general pregnant population. Similar information was described in the fluvoxamine prescription information, wherein there was a several-fold increase in the C_max_, AUC, and t_½_ in PM CYP2D6 compared to EM CYP2D6 [14]. Given the paucity of published data on fluvoxamine pharmacokinetics during pregnancy, stratified according to CYP2D6 phenotypes, the validated fluvoxamine model in healthy CYP2D6 subjects was used to support the exploration made regarding the pregnant population according to the CYP2D6 phenotype status, in addition to the validation of CYP2D6 abundance in pregnancy performed by Almurjan et al. [20] for the compound paroxetine.

#### 4.4.2. Maternal Plasma Concentration Changes throughout Pregnancy

The significant difference in both the trough and peak between the EM and PM phenotypes in healthy subjects is comparable to the pregnant population, as demonstrated in this study (Figure 10) [48,49]. The difference between UM and EM is significant for most of the GWs across three doses but with reduced magnitude when compared with the PM. Due to this difference, we explored the fluvoxamine dosage regimen for the UM population in pregnant women. This information can also be used to investigate the difference between the UM and EM CYP2D6 phenotypes in healthy subjects because of the guidance on dose selection for the CYP2D6 phenotypes provided by the Clinical Pharmacogenetics Implementation Consortium (CPIC) and the Dutch Pharmacogenetics Working Group (DPWG); however, there are no data for the UM CYP2D6 populations for any dose recommendations, unlike the EM and PM populations [86,87].

Regarding the plasma concentration trend throughout the gestational period within each CYP2D6 phenotype, it is comparable to the general pregnant population. The distinction between each phenotype mainly corresponded to when the difference became significant, which was earlier for both the UM and EM populations as it occurred at GW 5, whereas for PM, it occurred at GW 30. The percentage changes compared to baseline also showed the same pattern, which was higher in UM and EM at more than 50%, whereas for PM, this change was only around 25%. These results were anticipated since there is no functional allele in the PM CYP2D6 population, and the physiological and alternative clearance modification changes occurring throughout the gestational period constitute the primary factor that influences the concentration levels in the PM phenotype population [88,89,90]. As for the UM and EM populations, the trends are consistent with other drugs metabolised by the CYP2D6 enzyme, such as paroxetine, metoprolol, and codeine, because the abundance of the CYP2D6 enzyme increases throughout the gestational week up to the full term for both the UM and EM phenotypes [20,70,89,91].

#### 4.4.3. Umbilical Cord Concentration Changes throughout Pregnancy

No reported information (to date) is available to investigate the fluvoxamine foetal concentration based on the CYP2D6 phenotype. Thus, our simulation of the foetal concentration of fluvoxamine was based on the limited data used to validate the general fluvoxamine foetoplacental model. Since the foetoplacental model only focuses on the passive diffusion transfer mechanism through the placenta, the main potentiating factor in comparison between the UM, EM, and PM CYP2D6 phenotypes constitutes the CYP2D6 activities and physiological changes that occur throughout the gestational period. Generally, the factors that influence the crossing of a compound through the placenta are the physicochemical properties of the drug, physiological changes in the placenta such as blood flow, the involvement of active transport, and enzyme metabolism [92,93,94].

In the case of fluvoxamine, due to passive diffusion through lipid-soluble barriers of placental tissue membrane, cotyledons become the primary transfer pathway since fluvoxamine is a small molecule drug with base characteristics [92,93,95]. However, these characteristics did not differ between different CYP2D6 phenotypes. In addition, no data showed that fluvoxamine is transported by P-glycoproteins (P-gp), the major active efflux proteins for compound transport in the placenta [94]. Therefore, physiological changes and metabolism enzymes are the two main factors influencing fluvoxamine cord concentration.

The increasing trend in the fluvoxamine foetal concentrations in the PM population is solely due to the increase in placental blood flow throughout the gestational period since there are no active alleles of CYP2D6 [90,96]. In contrast, the cord concentration is consistent from GW 20 to full term for both the UM and EM populations, except for GW 30 for the peak of the EM population in all three doses. The consistent trend may be due to the counteractive effect that forms between an increase in the number of CYP2D6 metabolism enzymes in the mother and an increase in placenta blood flow towards full term [76,96]. The small but significant changes in the EM population for GW 30 may not be clinically significant since no evidence showed a direct correlation between foetal concentrations and the adverse reaction to the foetus [93,97]. Nevertheless, close monitoring may be needed, particularly when a high foetal concentration is expected.

#### 4.4.4. Fluvoxamine Dosing Adjustment during Pregnancy

This study identified that a dose increment is needed for the UM and EM populations to maintain a fluvoxamine maternal concentration within the therapeutic area (60–230 ng/mL). As for the PM population, a stable dose of 100 mg daily with an optional increase to 125 mg daily at GW 35 is sufficient to maintain a patient’s fluvoxamine concentration at the optimum level. The dosing recommendation is in line with the increasing CYP2D6 activity throughout the gestational period, which is the primary enzyme metabolising fluvoxamine [19,49,78]. Moreover, the 0.75- to 2-fold difference in suggested dosage between PM and UM as well as EM was anticipated since a dose reduction of 25–50% is recommended by Hicks et al. [86] for normal subjects. The widening of the dosage gap when approaching full term is in line with the increase in CYP2D6 activity throughout the gestational period [76].

The recommended dose for the UM and EM populations reached a maximum dose at GW 15 and GW 35, respectively (Table 8). The recommendation for a maximum dose in the UM population as early as GW 15 signalled the need for close monitoring, particularly with respect to TDM and clinical effects, in order to ensure that fluvoxamine is still effective in treating major depression, while a switch of antidepressant may also be considered, which is in line with the recommendations by Hicks et al. [86], Brouwer et al. [87], and Hiemke et al. [5]. In addition, the results align with the finding by Mulder et al. [98] in which a switch of antidepressants is more often needed in the UM than in the EM CYP2D6 population but not a change of dosing regimen. The need for increments up to the maximum dose in the UM/EM populations reflects the finding uncovered by Berard et al. [99], wherein the proportion of pregnant women with depression symptoms was higher in the UM/EM than in the PM CYP2D6 population even when treated with antidepressants.

Even though the recommended dose is lower in the PM population, the AUC is approximately twice that of the UM and EM populations, which is in stark contrast to the clearance value, where an increasing trend was seen in the UM and EM populations (Figure 12). Therefore, the risk for adverse events is higher in the PM population for both the mother and foetus since fluvoxamine accumulates in the body longer due to low clearance [100]. Dose adjustment, switching, and the discontinuation of antidepressants are frequently seen in the PM population due to adverse events, as it is difficult for pregnant women who may have already suffered from morning sickness to endure further adverse drug reactions [98,99,100,101].

A similar pattern was seen in the foetal concentration, which was higher in the PM population than in both the UM and EM populations, although with a lower recommended dose. The results from six studies showed that the rate of major congenital malformations and other adverse pregnancy outcomes did not differ significantly compared to the control groups [64,65,66,67,68,69]. Nevertheless, the number of subjects was still considered too small for any informed conclusions regarding usage during pregnancy to be made [102,103]. Moreover, the safety concern regarding the use of fluvoxamine as an SSRI is the potential risk of PPHN in the newborn [63,104]. Matsuoka et al. [85] have suggested dose tapering of 25 mg a week starting from GW 36 to reduce the risk of neonatal withdrawal syndrome, especially when the mother instantaneously discontinues the drug during pregnancy.

## 5. Conclusions

It is always a dilemma for a prescriber to decide between prescribing or withdrawing antidepressants during the perinatal period with respect to the health of both the mother and foetus. The prescriber’s main challenge is to find a balance between the treatment benefit throughout pregnancy and the risk of drug toxicity, particularly to the embryo and foetus. The physiological changes and those related to the biotransformation of metabolic enzymes during the gestational period are crucial factors in determining future actions regarding depression treatment in pregnant women. For fluvoxamine, the primary elimination route is through the CYP2D6 metabolism enzyme, which is highly polymorphic and, thus, further complicates the dosing strategies in pregnant women. Our developed models suggest that dose increments of fluvoxamine are needed among pregnant women, particularly for the UM and EM CYP2D6 populations. Although the fluvoxamine PBPK model developed in this study demonstrated a pragmatic method for determining a suitable dose in the perinatal setting, a confirmatory clinical trial is required to verify this study’s recommendations.

Even though TDM for the usage of antidepressants and phenotype testing before the initiation of SSRIs is not a regular practice in most clinical settings, this study highlighted the opportunity of using PBPK modelling for precision dosing, particularly in special populations such as pregnant women. The application of PBPK modelling combined with pre-emptive phenotyping may bring precision dosing closer to clinical settings, thereby improving the treatment of depression in the pregnant population.

## Figures and Tables

**Table 1:** Fluvoxamine compound parameters determined through a full PBPK model.

Parameters	Fluvoxamine	Notes
Compound type	Monoprotic Base	
Molecular weight (g/mol)	318.3	
Log P	3	
pKa 1	8.7	
fu	0.14	
B/P	0.826	Predicted in Simcyp^®^ based on Log P, plasma pH, haematocrit, and fu [36,37]
V_ss_ (L/kg)	35.48	Full PBPK model with K_p_ scalar of 13
K_p_	13	Estimated using Simcyp^®^ parameter estimation function
k_a_ (h^−1^)	0.15	Optimised through sensitivity analysis [14]
fa	0.8	Optimised through sensitivity analysis [14]
Lag time (h)	0	
Absorption Model	First Order	
Distribution Model	Full PBPK	
CL_PDM_ and CL_PDF_	0.253	Predicted from HBD and PSA information using [38]

Log P, partition coefficient; B/P, blood-to-plasma ratio; fu, unbound fraction; V_ss_, steady-state volume of distribution; K_p_, tissue partition coefficient; ka, absorption rate constant; fa, extent of absorption; CL_PDM_, maternal–placental permeability clearance; CL_PDF_, placental–foetal permeability clearance; HBD, hydrogen bond donor; PSA, polar surface area.

**Table 2:** Published data used in fluvoxamine model development and validation.

Study	Study Design	Number of Subjects	Age ^1^ (Years)	Dosing Regimen
Studies used for Model Development				
De Vries et al. [39]	Crossover with 7 days washout between each dose	12 healthy males	22–41	25 mg/50 mg/100 mg single-dose under fasted conditions
Van Harten et al. [40]	Crossover with 7 days washout between each period	8 healthy males and 4 healthy females	18–30	50 mg single-dose under fed and fasted conditions
Bahrami and Mohammadi [41]	Crossover bioequivalence study with 3 weeks washout period	24 healthy males	27.2 ± 3.1	100 mg single-dose
de Vries et al. [42]	Multiple-dose	3 healthy males and 3 healthy females	25–31	50 mg on day 1, followed by 50 mg twice daily from day 4 to day 31
Fleishaker and Hulst [43]	Multiple-dose	10 healthy males and 10 healthy females	20–44	50 mg daily for 3 days, followed by 100 mg daily for 7 days
Studies used for Model Validation				
Orlando et al. [44]	Single-dose	10 healthy males	35 ± 7	50 mg single-dose under fasted condition
Debree at al. [45]	Single-dose	9 healthy males and 1 healthy female	20–25	100 mg single-dose under fasted condition
USFDA [46]	Single-dose Study code: S1141107	15 healthy males and 13 healthy females	20.3–44.7	100 mg single-dose under fasted condition
Spigset et al. [47]	Multiple-dose	10 healthy males	28.9 ± 5.2	12.5 mg twice daily for 1st week, followed by 25 mg twice daily for 2nd week, 50 mg twice daily for 3rd week, and 100 mg twice daily for 4th week
USFDA [46]	Multiple-dose Study code: 1098001	12 healthy males with EM CYP2D6	19–43	100 mg daily for 10 days under fasting conditions
USFDA [46]	Multiple-dose Study code: 1098002	12 healthy males with EM CYP2D6	21–44	100 mg daily for 10 days under fasting conditions
Studies used for validation with CYP2D6 EM and PM population				
Carrillo et al. [48]	Single-dose	EM: 3 healthy males and 2 healthy females; PM: 2 healthy males and 1 healthy female	EM: 26–40 PM: 31–49	50 mg single-dose under fasting conditions
Spigset et al. [49]	Single-dose	EM: 7 healthy males and 3 healthy females; PM: 5 healthy males	EM: 28.7 ± 8.1 PM: 24.0 ± 1.6	50 mg single-dose under fasting conditions
Hartter et al. [50]	Single-dose	EM: 4 healthy males; PM: 1 healthy male	34–55	50 mg single-dose
Christensen et al. [51]	Single-dose and Multiple-dose	EM: 7 healthy subjects; PM: 5 healthy subjects	22–45	Period 1: EM—50 mg single-dose PM—25 mg single-dose Period 2: EM—25 mg twice daily for 7 days PM—25 mg daily for 7 days Period 3: EM—10 mg twice daily for 7 days PM—10 mg daily for 7 days

^1^ Age represented by range or mean ± SD.

**Table 3:** Pharmacokinetics of single- and multiple-dose studies (predicted and observed).

References	Dosing	PK Parameters	Observed	Predicted	Predicted/ Observed
Model Development					
			Geometric Mean (Range)		
De Vries et al. [39]	Single dose 25 mg	C_max_ (ng/mL)	8.80 (4.70–13.00)	7.77 (3.19–20.49)	0.88
		AUC_inf_ (ng/mL·h)	209.00 (117.00–425.00)	230.67 (97.76–571.74)	1.10
		T_max_ (h) ^1^	5.00 (1.00–8.00)	5.66 (3.40–13.35)	1.13
	Single dose 50 mg	C_max_ (ng/mL)	17.00 (8.40–28.00)	16.00 (6.39–40.97)	0.94
		AUC_inf_ (ng/mL·h)	448.00 (166.00–1115.00)	719.24 (254.16–3113.83)	1.61
		T_max_ (h) ^1^	4.80 (2.00–8.00)	5.67 (3.40–13.40)	1.03
	Single dose 100 mg	C_max_ (ng/mL)	36.00 (21.00–60.00)	32.01 (12.78–81.95)	0.89
		AUC_inf_ (ng/mL·h)	927.00 (325.00–2146.00)	1693.24 (585.86–10825.67)	1.83
		T_max_ (h) ^1^	4.50 (3.00–6.00)	5.68 (3.40–13.35)	1.04
Van Harten et al. [40]	Single dose 50 mg—Fast	C_max_ (ng/mL)	15.40 (7.50–27.00)	16.00 (6.39–40.97)	1.04
		AUC_0–32 h_ (ng/mL·h)	237.00 (102.00–571.00)	324.19 (139.89–710.72)	1.37
		T_max_ (h) ^1^	6.00 (3.00–12.00)	5.67 (3.40–13.35)	0.95
	Single dose 50 mg—Fed	C_max_ (ng/mL)	15.50 (10.00–32.00)	16.00 (6.39–40.97)	1.03
		AUC_0–32 h_ (ng/mL·h)	223.00 (65.00–587.00)	324.19 (139.89–710.72)	1.45
		T_max_ (h) ^1^	7.00 (2.00–12.00)	5.67 (3.40–13.35)	0.81
de Vries et al. [42]	Single dose 50 mg—Day 1	C_max_ (ng/mL)	30.00 (13.10)	17.82 (9.63)	0.59
		AUC_inf_ (ng/mL·h)	652.00 (319.00)	882.08 (717.30)	1.35
		T_max_ (h)^1^	6.00 (4.00–8.00)	5.63 (3.25–14.50)	0.94
	Multiple doses of 50 mg twice daily from Day 4 to Day 31	C_max_ (ng/mL)	93.00 (96.16)	81.55 (60.34)	0.88
		AUC_0–12 h_ (ng/mL·h)	873.00 (782.44)	920.77 (707.31)	1.05
		T_max_ (h) ^1^	5.00 (1.00–10.00)	3.48 (2.65–4.20)	0.70
			Arithmetic Mean (SD)		
Fleishaker and Hulst [43]	Single dose 50 mg—Day 1	C_max_ (ng/mL)	21.50 (4.89)	16.77 (6.70)	0.78
		AUC_0–24 h_ (ng/mL·h)	328.00 (84.60)	283.65 (107.45)	0.86
		T_max_ (h)	5.70 (1.49)	5.67 (1.45)	0.99
	Multiple doses of 50 mg daily for 3 days followed by 100 mg daily for the 7 days	C_max_ (ng/mL)	99.30 (35.00)	80.32 (36.45)	0.81
		AUC_0–24 h_ (ng/mL·h)	1762.00 (737.00)	1614.20 (786.04)	0.92
		T_max_ (h)	7.95 (4.91)	4.75 (0.78)	0.60
Bahrami and Mohammadi [41]	Single dose 100 mg—Test	C_max_ (ng/mL)	46.20 (29.00)	34.65 (13.94)	0.75
		AUC_0–48 h_ (ng/mL·h)	866.20 (480.00)	872.88 (351.91)	1.01
		AUC_inf_ (ng/mL·h)	1308.00 (781.00)	1641.58 (902.86)	1.26
		T_max_ (h)	5.30 (2.00)	5.68 (1.45)	1.07
	Single dose 100 mg—Reference	C_max_ (ng/mL)	48.50 (28.00)	34.65 (13.94)	0.71
		AUC_0–48 h_ (ng/mL·h)	802.20 (360.00)	872.88 (351.91)	1.09
		AUC_inf_ (ng/mL·h)	1224.90 (430.00)	1641.58 (902.86)	1.34
		T_max_ (h)	5.60 (2.10)	5.68 (1.45)	1.01
Model Validation					
			Arithmetic Mean (SD)		
Orlando et al. [44]	Single dose 50 mg	C_max_ (ng/mL)	15.00 (3.00)	17.32 (6.97)	1.15
		AUC_inf_ (ng/mL·h)	304.00 (84.00)	820.98 (452.53)	2.70
		T_max_ (h) ^2^	5.00 (4.00–8.00)	5.47 (3.40–13.40)	1.08
			Geometric Mean (SD)		
Debree et al. [45]	Single dose 100 mg	C_max_ (ng/mL)	49.30 (17.00)	32.01 (13.94)	0.65
		AUC_0–24 h_ (ng/mL·h)	523.90 (122.90)	545.44 (228.11)	1.04
		AUC_inf_ (ng/mL·h)	817.00 (194.30)	958.18 (472.25)	1.17
		T_max_ (h) ^1^	5.00 (2.00–8.00)	5.68 (3.40–13.40)	1.14
			Arithmetic Mean (SD)		
USFDA [46]	Single dose 100 mg	C_max_ (ng/mL)	41.88 (18.99)	34.65 (13.94)	0.83
		AUC_inf_ (ng/mL·h)	959.33 (520.71)	2071.01 (1435.11)	2.16
		T_max_ (h) ^1^	6.00 (4.00–16.00)	5.68 (3.40–13.35)	0.95
Spigset et al. [47]	Week 1—12.5 mg twice daily for 7 days	C_max_ (nmol/L)	25.10 (9.40)	57.96 (29.62)	2.31
		AUC_12 h_ (nmol.hr/L)	236.00 (95.00)	652.00 (339.68)	2.76
	Week 1—25 mg twice daily for 7 days	C_max_ (nmol/L)	76.30 (22.10)	107.53 (60.63)	1.41
		AUC_12 h_ (nmol.hr/L)	745.00 (258.00)	1457.38 (795.37)	1.96
	Week 1—50 mg twice daily for 7 days	C_max_ (nmol/L)	244.00 (97.90)	261.50 (141.94)	1.07
		AUC_12 h_ (nmol/L·hr)	2391.00 (949.00)	2960.86 (1643.50)	1.24
	Week 1—100 mg twice daily for 7 days	C_max_ (nmol/L)	738.00 (314.00)	439.88 (254.36)	0.60
		AUC_12 h_ (nmol·hr/L)	7545.00 (3239.00)	5943.80 (3317.30)	0.79
USFDA [46]	Multiple doses of 100 mg daily for 10 days (Prot. C)	C_max_ (ng/mL)	107.00 (73.52)	79.41 (31.39)	0.74
		AUC_0–24 h_ (ng/mL·h)	1738.55 (1392.42)	1587.74 (669.76)	0.91
	Multiple doses of 100 mg daily for 10 days (Prot. D)	C_max_ (ng/mL)	129.59 (62.86)	85.97 (42.84)	0.66
		AUC_0–24 h_ (ng/mL·h)	2109.30 (1085.63)	1677.97 (905.71)	0.80
Model validation for EM and PM CYP2D6 Phenotype population					
Carrillo et al. [48]	Single dose 50 mg -EM CYP2D6	C_max_ (nmol/L)	85.90 (42.50)	61.03 (28.98)	0.71
		AUC_0–32 h_ (nmol/L·h) ^3^	1097.90 (180.35)	1220.62 (55.36)	1.11
		AUC_inf_ (nmol/L·h)	1352.00 (733.00)	2065.41 (1033.85)	1.53
		T_max_ (h)	4.4 (2.1)	5.57 (1.52)	1.27
	Single dose 50 mg -PM CYP2D6	C_max_ (nmol/L)	178.10 (27.50)	78.81 (33.53)	0.44
		AUC_0–72 h_ (nmol/L·h) ^3^	4648.59 (237.46)	2889.62 (118.60)	0.62
		AUC_inf_ (nmol/L·h)	5290.00 (332.00)	6287.38 (2990.77)	1.19
		T_max_ (h)	4.60 (2.30)	7.01 (1.82)	1.52
Spigset et al. [49]	Single dose 50 mg -EM CYP2D6	C_max_ (nmol/L)	44.50 (12.30)	61.03 (28.98)	1.37
		AUC_0–48 h_ (nmol/L·h) ^3^	870.00 (110.00)	1530.00 (70.00)	1.76
		AUC_inf_ (nmol/L·h)	1000.00 (410.00)	2610.00 (1280.00)	2.61
		T_max_ (h)	7.80 (2.40)	5.57 (1.52)	0.71
	Single dose 50 mg -PM CYP2D6	C_max_ (nmol/L)	50.40 (17.80)	78.81 (33.53)	1.56
		AUC_0–48 h_ (nmol/L·h) ^3^	1090.00 (160.00)	2280.00 (90.00)	2.09
		AUC_inf_ (nmol/L·h)	1310.00 (670.00)	4950.00 (2220.00)	3.78
		T_max_ (h)	6.60 (2.10)	7.01 (1.82)	1.06
Hartter et al. [50]	Single dose 50 mg -EM CYP2D6	C_max_ (ng/mL)	13.00 (3.7)	19.43 (9.22)	1.49
		AUC_0–28 h_ (mcg/L·h)	185.50 (33.60)	360.58 (163.21)	1.94
	Single dose 50 mg -PM CYP2D6 ^4^	C_max_ (ng/mL)	48.00	25.09 (10.67)	0.52
		AUC_0–28 h_ (mcg/L·h)	612.00	512.06 (206.59)	0.84
Chtistensen et al. [51]	Single dose 50 mg -EM CYP2D6	C_max_ (nmol/L)	58.14 (37.90)	61.03 (28.98)	1.05
	Single dose 25 mg -PM CYP2D6	C_max_ (nmol/L)	23.20 (2.28)	39.41 (16.76)	1.70

AUC_inf_, area-under-the-curve to infinity; AUC_0-t_, area-under-the-curve to the last time point; AUC_t_, area-under-the-curve for the total hour at steady-state; C_max_, maximum plasma concentration; Tmax, time to reach maximum plasma concentration. ^1^—Arithmetic mean (range); ^2^—median; ^3^—the AUC_0-t_ was calculated from the published graph; ^4^—only 1 subject, so no standard deviation was reported.

**Table 4:** Predicted fluvoxamine plasma-concentration across the maternity period.

Pharmacokinetic Metric	Gestational Week (GW)								
	0 (Baseline)	5	10	15	20	25	30	35	40 (Full Term)
Steady-state C_min_ (ng/mL)	75.23 (52.73)	76.34 (56.97)	71.37 (55.5)	65.72 (53.73)	59.84 (51.76)	54.04 (49.62)	48.52 (47.31)	43.32 (44.8)	38.4 (42.05)
% change from baseline	0	1.48	−5.13	−12.64	−20.46	−28.17	−35.50	−42.42	−48.96
*p*-value ^1^		0.9998	0.9966	0.6707	0.1718	0.0215	0.0016	<0.0001	<0.0001
% C_min_ < 60 ng/mL ^2^ (%)	46	48	54	58	66	68	74	80	85
Steady-state C_max_ (ng/mL)	112.5 (64.17)	113.1 (67.3)	106.1 (65.56)	97.87 (63.45)	89.09 (61.06)	80.29 (58.43)	71.85 (55.61)	64.03 (52.63)	56.96 (49.53)
% change from baseline	0	0.53	−5.69	−13.00	−20.81	−28.63	−36.13	−43.08	−49.37
*p*-value ^1^		>0.9999	0.9739	0.3875	0.0385	0.0012	<0.0001	<0.0001	<0.0001
% C_max_ < 60 ng/mL ^2^ (%)	17	20	23	29	38	45	56	60	67

C_min_, trough plasma-concentration; C_max_, maximum plasma-concentration; ^1^ *p*-value: statistical significance test between each GW with baseline; ^2^ Efficacy threshold (60–230 ng/mL) as recommended in Consensus Guidelines for Therapeutic Drug Monitoring in Neuropsychopharmacology: Update 2017 [5]; Mean (SD).

**Table 5:** Summary of predicted fluvoxamine plasma concentrations during gestation.

			Gestational Week (GW)								
Daily Dose	Phenotype	Pharmacokinetic Metric	0 (Baseline)	5	10	15	20↓	25	30	35	40 (Full Term)
50 mg	UM CYP2D6	Steady-state C_min_ (ng/mL)	20.34 (11.28)	20.19 (13.18)	18.19 (12.16)	16.04 (10.99)	15.90 (11.52)	13.43 (9.68)	11.40 (8.40)	9.61 (7.21)	8.05 (6.15)
		% change from baseline		−0.72	−10.55	−21.13	−21.82	−33.94	−43.93	−52.75	−60.40
		*p*-value ^1^		0.9999	0.5661	0.0229	0.0171	<0.0001	<0.0001	<0.0001	<0.0001
		% C_min_ < 60 ng/mL ^2^	100	98	98	99	100	100	100	100	100
		Steady-state C_max_ (ng/mL)	35.82 (16.28)	35.09 (17.59)	31.98 (16.38)	28.49 (14.99)	28.57 (16.75)	24.98 (14.64)	21.80 (13.08)	18.86 (11.57)	16.20 (10.16)
		% change from baseline		−2.04	−10.72	−20.47	−20.24	−30.27	−39.15	−47.34	−54.77
		*p*-value ^1^		0.9995	0.3221	0.0037	0.0043	<0.0001	<0.0001	<0.0001	<0.0001
		% C_max_ > 230 ng/mL^2^	0	0	0	0	0	0	0	0	0
	EM CYP2D6	Steady-state C_min_ (ng/mL)	30.14 (19.33)	28.07 (16.06)	25.80 (15.07)	23.27 (13.90)	22.36 (14.29)	19.69 (12.66)	17.00 (11.05)	14.54 (9.56)	12.36 (8.20)
		% change from baseline		−6.87	−14.40	−22.80	−25.82	−34.67	−43.61	−51.75	−59.01
		*p*-value ^1^		0.8536	0.1452	0.0033	0.0006	<0.0001	<0.0001	<0.0001	<0.0001
		% C_min_ < 60 ng/mL ^2^	93	95	98	98	97	98	99	100	100
		Steady-state C_max_ (ng/mL)	46.99 (23.79)	44.73 (20.46)	41.44 (19.29)	37.65 (17.91)	36.64 (18.90)	32.91 (17.07)	29.11 (15.27)	25.53 (13.55)	22.20 (11.94)
		% change from baseline		−4.81	−11.80	−19.89	−22.02	−29.96	−38.04	−45.68	−52.76
		*p*-value ^1^		0.9358	0.1602	0.0019	0.0004	<0.0001	<0.0001	<0.0001	<0.0001
		% C_max_ > 230 ng/mL ^2^	0	0	0	0	0	0	0	0	0
	PM CYP2D6	Steady-state C_min_ (ng/mL)	68.51 (36.54)	67.54 (32.65)	66.21 (31.92)	64.58 (30.97)	60.80 (29.70)	57.69 (26.25)	55.29 (25.09)	52.68 (23.83)	49.88 (22.49)
		% change from baseline		−1.43	−3.37	−5.75	−11.26	−15.80	−19.30	−23.10	−27.19
		*p*-value ^1^		0.9997	0.9944	0.9102	0.302	0.0564	0.0102	0.0011	<0.0001
		% C_min_ < 60 ng/mL ^2^	44	45	48	50	58	60	65	65	69
		Steady-state C_max_ (ng/mL)	90.43 (40.94)	89.40 (37.15)	87.17 (36.23)	84.38 (35.04)	80.17 (34.19)	76.21 (30.35)	73.03 (29.04)	69.54 (27.62)	65.77 (26.08)
		% change from baseline		−1.14	−3.60	−6.68	−11.34	−15.72	−19.24	−23.10	−27.27
		*p*-value ^1^		0.9997	0.9848	0.7071	0.164	0.0184	0.0018	<0.0001	<0.0001
		% C_max_ > 230 ng/mL^2^	2	1	1	1	1	0	0	0	0
100 mg	UM CYP2D6	Steady-state C_min_ (ng/mL)	40.70 (22.58)	40.41 (26.40)	36.41 (24.34)	32.11 (22.01)	31.82 (23.08)	26.89 (19.39)	22.82 (16.82)	19.23 (14.45)	16.11 (12.31)
		% change from baseline		−0.72	−10.55	−21.13	−21.82	−33.94	−43.93	−52.75	−60.41
		*p*-value ^1^		0.9999	0.5666	0.023	0.0172	<0.0001	<0.0001	<0.0001	<0.0001
		% C_min_ < 60 ng/mL ^2^	78	78	84	92	90	94	94	97	99
		Steady-state C_max_ (ng/mL)	71.70 (32.59)	70.23 (35.22)	64.01 (32.80)	57.02 (30.01)	57.18 (33.54)	49.99 (29.32)	43.63 (26.19)	37.75 (23.18)	32.42 (20.34)
		% change from baseline		−2.04	−10.72	−20.46	−20.24	−30.27	−39.15	−47.34	−54.77
		*p*-value ^1^		0.9995	0.3225	0.0038	0.0043	<0.0001	<0.0001	<0.0001	<0.0001
		% C_max_ > 230 ng/mL ^2^	0	0	0	0	0	0	0	0	0
	EM CYP2D6	Steady-state C_min_ (ng/mL)	60.33 (38.70)	56.19 (32.17)	51.64 (30.17)	46.57 (27.84)	44.76 (28.65)	39.42 (25.37)	34.03 (22.14)	29.11 (19.15)	24.73 (16.43)
		% change from baseline		−6.87	−14.40	−22.81	−25.82	−34.66	−43.60	−51.75	−59.01
		*p*-value ^1^		0.8538	0.1455	0.0033	0.0006	<0.0001	<0.0001	<0.0001	<0.0001
		% C_min_ < 60 ng/mL ^2^	60	62	72	78	78	83	89	96	97
		Steady-state C_max_ (ng/mL)	94.04 (47.64)	89.52 (40.96)	82.95 (38.62)	75.34 (35.85)	73.35 (37.87)	65.87 (34.20)	58.27 (30.60)	51.09 (27.15)	44.43 (23.91)
		% change from baseline		−4.81	−11.80	−19.89	−22.01	−29.96	−38.04	−45.67	−52.75
		*p*-value ^1^		0.9359	0.1607	0.0019	0.0004	<0.0001	<0.0001	<0.0001	<0.0001
		% C_max_ > 230 ng/mL ^2^	2	1	1	1	1	0	0	0	0
	PM CYP2D6	Steady-state C_min_ (ng/mL)	137.03 (73.09)	135.07 (65.31)	132.41 (63.84)	129.15 (61.95)	121.60 (59.40)	115.38 (52.50)	110.58 (50.17)	105.37 (47.66)	99.77 (44.98)
		% change from baseline		−1.43	−3.37	−5.75	−11.26	−15.80	−19.30	−23.10	−27.19
		*p*-value ^1^		0.9997	0.9944	0.9103	0.302	0.0565	0.0102	0.0011	<0.0001
		% C_min_ < 60 ng/mL ^2^	7	7	7	7	11	11	13	17	19
		Steady-state C_max_ (ng/mL)	180.86 (81.88)	178.80 (74.3)	174.34 (72.45)	168.77 (70.08)	160.34 (68.38)	152.43 (60.71)	146.05 (58.08)	139.08 (55.23)	131.54 (52.17)
		% change from baseline		−1.14	−3.60	−6.69	−11.34	−15.72	−19.24	−23.10	−27.27
		*p*-value ^1^		0.9997	0.9848	0.707	0.1641	0.0184	0.0018	<0.0001	<0.0001
		% C_max_ > 230 ng/mL ^2^	22	18	15	13	16	7	4	4	4
300 mg	UM CYP2D6	Steady-state C_min_ (ng/mL)	146.24 (76.37)	144.43 (87.69)	130.69 (81.21)	115.76 (73.81)	114.73 (77.53)	97.84 (65.77)	83.65 (57.51)	70.98 (49.80)	59.88 (42.77)
		% change from baseline		−1.24	−10.63	−20.84	−21.55	−33.09	−42.80	−51.46	−59.06
		*p*-value ^1^		0.9997	0.4863	0.0144	0.0103	<0.0001	<0.0001	<0.0001	<0.0001
		% C_min_ < 60 ng/mL ^2^	9	13	15	21	28	32	38	51	65
		Steady-state C_max_ (ng/mL)	184.13 (87.29)	180.98 (97.07)	164.56 (90.20)	146.38 (82.30)	146.20 (89.44)	126.68 (77.14)	109.74 (68.24)	94.32 (59.82)	80.52 (52.01)
		% change from baseline		−1.72	−10.63	−20.50	−20.60	−31.20	−40.40	−48.78	−56.27
		*p*-value ^1^		0.9996	0.378	0.0062	0.0058	<0.0001	<0.0001	<0.0001	<0.0001
		% C_max_ > 230 ng/mL ^2^	28	31	22	13	15	10	6	6	2
	EM CYP2D6	Steady-state C_min_ (ng/mL)	209.09 (124.25)	196.46 (103.95)	181.21 (97.8)	164.03 (90.56)	157.89 (95.00)	139.86 (84.78)	121.61 (74.65)	104.83 (65.14)	89.70 (56.41)
		% change from baseline		−6.04	−13.33	−21.55	−24.48	−33.11	−41.84	−49.86	−57.10
		*p*-value ^1^		0.8938	0.1619	0.0034	0.0006	<0.0001	<0.0001	<0.0001	<0.0001
		% C_min_ < 60 ng/mL ^2^	5	3	4	5	11	13	20	25	38
		Steady-state C_max_ (ng/mL)	249.43 (134.54)	236.20 (114.15)	218.50 (107.52)	198.28 (99.70)	192.26 (105.20)	171.75 (94.46)	150.93 (83.85)	131.50 (73.82)	113.68 (64.51)
		% change from baseline		−5.30	−12.40	−20.51	−22.92	−31.14	−39.49	−47.28	−54.42
		*p*-value ^1^		0.917	0.1583	0.0023	0.0005	<0.0001	<0.0001	<0.0001	<0.0001
		% C_max_ > 230 ng/mL ^2^	50	47	37	27	25	21	16	9	3
	PM CYP2D6	Steady-state C_min_ (ng/mL)	446.83 (223.35)	441.25 (200.60)	432.13 (196.01)	420.84 (190.08)	396.98 (182.97)	377.01 (161.66)	361.44 (154.58)	344.51 (146.93)	326.3 (138.77)
		% change from baseline		−1.25	−3.29	−5.82	−11.16	−15.63	−19.11	−22.90	−26.97
		*p*-value ^1^		0.9997	0.9936	0.8757	0.2526	0.0398	0.006	0.0005	<0.0001
		% C_min_ < 60 ng/mL ^2^	0	0	0	0	0	0	0	0	0
		Steady-state C_max_ (ng/mL)	500.13 (236.64)	494.14 (213.61)	482.70 (208.5)	468.46 (201.93)	443.69 (195.69)	421.55 (173.34)	403.91 (165.78)	384.70 (157.60)	363.99 (148.84)
		% change from baseline		−1.20	−3.49	−6.33	−11.28	−15.71	−19.24	−23.08	−27.22
		*p*-value ^1^		0.9997	0.9902	0.7867	0.1985	0.0257	0.0031	0.0002	<0.0001
		% C_max_ > 230 ng/mL ^2^	94	93	93	93	90	89	87	86	81

C_min_, trough plasma concentration; C_max_, maximum plasma concentration; ^1^ *p*-value: statistical significance test between each GW with respect to baseline; ^2^ efficacy threshold (60–230 ng/mL) as recommended in Consensus Guidelines for Therapeutic Drug Monitoring in Neuropsychopharmacology: Update 2017 [5]; mean (SD); ↓, initiation of foetoplacental PBPK model.

**Table 6:** Summary of simulated fluvoxamine umbilical cord concentrations during gestation.

			Gestational Week (GW)				
Daily Dose	Phenotype	Pharmacokinetic Metric	20	25	30	35	40 (Full Term)
50 mg	UM CYP2D6	Steady-state C_min_ (ng/mL)	8.60 (6.46)	9.61 (7.34)	9.84 (7.70)	9.54 (7.63)	8.98 (7.31)
		% change from GW-20		11.69	14.40	10.96	4.43
		*p*-value *		0.7359	0.5769	0.7761	0.9888
		Steady-state C_max_ (ng/mL)	15.36 (9.22)	17.65 (10.80)	18.35 (11.55)	17.88 (11.56)	16.8 (11.13)
		% change from GW-20		14.91	19.48	16.42	9.39
		*p*-value *		0.3882	0.1715	0.3036	0.7637
	EM CYP2D6	Steady-state C_min_ (ng/mL)	11.90 (8.25)	13.76 (9.60)	14.28 (10.08)	14.02 (9.99)	13.34 (9.58)
		% change from GW-20		15.71	20.08	17.88	12.13
		*p*-value *		0.4456	0.2287	0.3151	0.6341
		Steady-state C_max_ (ng/mL)	19.37 (10.81)	22.79 (12.78)	24 (13.63)	23.71 (13.64)	22.58 (13.16)
		% change from GW-20		17.67	23.92	22.42	16.58
		*p*-value *		0.1931	0.041	0.0594	0.2244
	PM CYP2D6	Steady-state C_min_ (ng/mL)	33.00 (16.85)	40.91 (19.52)	47.10 (22.46)	51.39 (24.44)	54.31 (25.74)
		% change from GW-20		23.95	42.72	55.71	64.58
		*p*-value *		0.0424	<0.0001	<0.0001	<0.0001
		Steady-state C_max_ (ng/mL)	43.22 (19.21)	53.59 (22.26)	61.42 (25.56)	66.47 (27.65)	69.54 (28.91)
		% change from GW-20		23.98	42.11	53.78	60.88
		*p*-value *		0.0139	<0.0001	<0.0001	<0.0001
100 mg	UM CYP2D6	Steady-state C_min_ (ng/mL)	17.22 (12.94)	19.23 (14.69)	19.69 (15.43)	19.10 (15.28)	17.98 (14.63)
		% change from GW-20		11.68	14.39	10.95	4.41
		*p*-value *		0.7365	0.5781	0.777	0.9889
		Steady-state C_max_ (ng/mL)	30.75 (18.46)	35.33 (21.62)	36.73 (23.13)	35.8 (23.15)	33.63 (22.29)
		% change from GW-20		14.90	19.47	16.42	9.38
		*p*-value *		0.3889	0.1721	0.3042	0.7647
	EM CYP2D6	Steady-state C_min_ (ng/mL)	23.81 (16.54)	27.55 (19.23)	28.59 (20.2)	28.07 (20.02)	26.70 (19.20)
		% change from GW-20		15.71	20.07	17.86	12.11
		*p*-value *		0.4463	0.2297	0.3164	0.6357
		Steady-state C_max_ (ng/mL)	38.77 (21.65)	45.63 (25.60)	48.05 (27.31)	47.47 (27.33)	45.20 (26.36)
		% change from GW-20		17.67	23.92	22.42	16.58
		*p*-value *		0.1935	0.0412	0.0598	0.2253
	PM CYP2D6	Steady-state C_min_ (ng/mL)	66.00 (33.70)	81.81 (39.04)	94.20 (44.93)	102.77 (48.89)	108.63 (51.48)
		% change from GW-20		23.95	42.72	55.71	64.58
		*p*-value *		0.0425	<0.0001	<0.0001	<0.0001
		Steady-state C_max_ (ng/mL)	86.44 (38.42)	107.18 (44.52)	122.84 (51.12)	132.93 (55.31)	139.06 (57.81)
		% change from GW-20		23.98	42.11	53.78	60.87
		*p*-value *		0.0139	<0.0001	<0.0001	<0.0001
300 mg	UM CYP2D6	Steady-state C_min_ (ng/mL)	62.05 (43.4)	69.96 (49.76)	72.27 (52.72)	70.72 (52.68)	67.06 (50.82)
		% change from GW-20		12.75	16.48	13.98	8.08
		*p*-value *		0.6345	0.4114	0.5583	0.892
		Steady-state C_max_ (ng/mL)	78.68 (49.54)	89.65 (57.41)	92.71 (61.00)	90.21 (60.84)	84.94 (58.56)
		% change from GW-20		13.95	17.84	14.66	7.96
		*p*-value *		0.4771	0.2612	0.4329	0.8636
	EM CYP2D6	Steady-state C_min_ (ng/mL)	83.97 (54.74)	97.78 (64.14)	102.33 (67.94)	101.33 (67.89)	97.10 (65.53)
		% change from GW-20		16.45	21.87	20.67	15.63
		*p*-value *		0.3650	0.1401	0.1708	0.3843
		Steady-state C_max_ (ng/mL)	101.77 (60.36)	119.11 (70.95)	124.76 (75.21)	123.01 (75.01)	117.28 (72.28)
		% change from GW-20		17.04	22.59	20.87	15.24
		*p*-value *		0.2577	0.0774	0.112	0.3306
	PM CYP2D6	Steady-state C_min_ (ng/mL)	215.23 (103.83)	267.09 (120.31)	307.70 (138.54)	335.79 (150.74)	354.74 (158.57)
		% change from GW-20		24.09	42.96	56.01	64.82
		*p*-value *		0.0274	<0.0001	<0.0001	<0.0001
		Steady-state C_max_ (ng/mL)	239.62 (110.43)	296.94 (127.87)	340.49 (146.82)	369.09 (159.08)	387.32 (166.69)
		% change from GW-20		23.92	42.10	54.03	61.64
		*p*-value *		0.0194	<0.0001	<0.0001	<0.0001

C_min_, trough umbilical cord concentration; C_max_, maximum umbilical cord concentration; * *p*-value: statistical significance test between each GW with GW-20; mean (SD).

**Table 7:** Percentage of subjects with trough and peak outside the therapeutic window.

Phenotype	Dose	Pharmacokinetic Metric	Gestational Week								
			0	5	10	15	20	25	30	35	40
UM CYP2D6	50 mg	C_min_ < 60 ng/mL	100	98	98	99	100	100	100	100	100
		C_max_ > 230 ng/mL	0	0	0	0	0	0	0	0	0
	75 mg	C_min_ < 60 ng/mL	94	94	96	97	93	97	98	100	100
		C_max_ > 230 ng/mL	0	0	0	0	0	0	0	0	0
	100 mg	C_min_ < 60 ng/mL	78	78	84	92	90	94	94	97	99
		C_max_ > 230 ng/mL	0	0	0	0	0	0	0	0	0
	125 mg	C_min_ < 60 ng/mL	67	67	72	79	81	89	94	94	95
		C_max_ > 230 ng/mL	0	2	1	0	1	0	0	0	0
	150 mg	C_min_ < 60 ng/mL	55	61	66	70	72	82	89	94	94
		C_max_ > 230 ng/mL	1	2	2	2	1	1	1	0	0
	175 mg	C_min_ < 60 ng/mL	40	46	50	58	55	67	74	80	88
		C_max_ > 230 ng/mL	1	2	2	2	2	1	0	0	0
	200 mg	C_min_ < 60 ng/mL	29	32	37	49	40	53	67	74	80
		C_max_ > 230 ng/mL	7	5	3	2	5	2	1	0	0
	225 mg	C_min_ < 60 ng/mL	27	27	32	43	37	49	58	68	77
		C_max_ > 230 ng/mL	8	8	6	3	7	4	2	1	0
	250 mg	C_min_ < 60 ng/mL	15	16	25	32	33	38	51	64	72
		C_max_ > 230 ng/mL	15	14	8	7	9	6	2	2	1
	275 mg	C_min_ < 60 ng/mL	11	15	20	28	30	37	43	55	68
		C_max_ > 230 ng/mL	24	22	14	8	11	6	6	2	1
	300 mg	C_min_ < 60 ng/mL	9	13	15	21	28	32	38	51	65
		C_max_ > 230 ng/mL	28	31	22	13	15	10	6	6	2
EM CYP2D6	50 mg	C_min_ < 60 ng/mL	93	95	98	98	97	98	99	100	100
		C_max_ > 230 ng/mL	0	0	0	0	0	0	0	0	0
	75 mg	C_min_ < 60 ng/mL	80	86	90	92	91	96	97	97	98
		C_max_ > 230 ng/mL	1	0	0	0	0	0	0	0	0
	100 mg	C_min_ < 60 ng/mL	60	62	72	78	78	83	89	96	97
		C_max_ > 230 ng/mL	2	1	1	1	1	0	0	0	0
	125 mg	C_min_ < 60 ng/mL	40	46	53	61	61	74	80	84	93
		C_max_ > 230 ng/mL	4	3	3	2	3	2	0	0	0
	150 mg	C_min_ < 60 ng/mL	32	34	41	48	50	58	73	79	83
		C_max_ > 230 ng/mL	8	4	4	3	3	3	3	1	0
	175 mg	C_min_ < 60 ng/mL	19	13	19	24	35	41	46	55	74
		C_max_ > 230 ng/mL	9	6	5	4	3	3	3	2	0
	200 mg	C_min_ < 60 ng/mL	12	7	10	16	22	34	42	46	55
		C_max_ > 230 ng/mL	16	12	8	6	5	3	3	3	2
	225 mg	C_min_ < 60 ng/mL	10	5	7	10	20	24	36	42	48
		C_max_ > 230 ng/mL	22	15	12	9	10	5	3	3	3
	250 mg	C_min_ < 60 ng/mL	7	5	5	7	15	20	25	39	43
		C_max_ > 230 ng/mL	34	24	17	14	17	13	5	3	3
	275 mg	C_min_ < 60 ng/mL	6	4	5	5	12	16	22	36	42
		C_max_ > 230 ng/mL	45	34	26	17	21	15	11	3	3
	300 mg	C_min_ < 60 ng/mL	5	3	4	5	11	13	20	25	38
		C_max_ > 230 ng/mL	50	47	37	27	25	21	16	9	3
PM CYP2D6	50 mg	C_min_ < 60 ng/mL	44	45	48	50	58	60	65	65	69
		C_max_ > 230 ng/mL	2	1	1	1	1	0	0	0	0
	75 mg	C_min_ < 60 ng/mL	18	19	20	21	23	26	30	33	37
		C_max_ > 230 ng/mL	4	4	4	4	3	3	3	3	2
	100 mg	C_min_ < 60 ng/mL	7	7	7	7	11	11	13	17	19
		C_max_ > 230 ng/mL	22	18	15	13	16	7	4	4	4
	125 mg	C_min_ < 60 ng/mL	2	2	2	3	4	5	6	8	9
		C_max_ > 230 ng/mL	45	47	44	43	29	24	21	19	16
	150 mg	C_min_ < 60 ng/mL	1	2	2	2	2	2	2	3	4
		C_max_ > 230 ng/mL	64	65	59	57	46	45	44	36	28
	175 mg	C_min_ < 60 ng/mL	0	0	0	0	0	0	0	1	1
		C_max_ > 230 ng/mL	65	68	68	65	56	47	45	41	37
	200 mg	C_min_ < 60 ng/mL	0	0	0	0	0	0	0	0	0
		C_max_ > 230 ng/mL	76	73	72	72	67	65	60	57	47
	225 mg	C_min_ < 60 ng/mL	0	0	0	0	0	0	0	0	0
		C_max_ > 230 ng/mL	81	78	77	76	73	69	69	66	61
	250 mg	C_min_ < 60 ng/mL	0	0	0	0	0	0	0	0	0
		C_max_ > 230 ng/mL	85	87	86	82	81	79	76	72	69
	275 mg	C_min_ < 60 ng/mL	0	0	0	0	0	0	0	0	0
		C_max_ > 230 ng/mL	91	93	91	89	86	86	82	79	74
	300 mg	C_min_ < 60 ng/mL	0	0	0	0	0	0	0	0	0
		C_max_ > 230 ng/mL	94	93	93	93	90	89	87	86	81

C_min_, trough umbilical cord concentration; C_max_, maximum umbilical cord concentration; the red column indicates the percentage of subjects where the trough and peak outside the therapeutic window (60–230 ng/mL) is more than 20%.

**Table 8:** Summary of recommended daily dose, predicted clearance, area-under-the-curve, and umbilical cord concentrations based on the recommended doses.

			Gestational Week (GW)								
Phenotype	Pharmacokinetic Metric		0 (Baseline)	5	10	15	20↓	25	30	35	40 (Full Term)
UM CYP2D6	Recommended daily dose (mg)		250	250	275	300	300	300	300	300	300
	CL (L/h)		95.69 (51.51)	101.60 (59.00)	104.50 (62.05)	128.70 (77.43)	144.40 (113.00)	166.70 (131.00)	196.90 (157.80)	234.20 (190.80)	279.90 (231.30)
	AUC (ng/mL·h)		1691.00 (836.10)	1665.00 (944.40)	1640.00 (958.40)	1611.00 (959.20)	1603.00 (1025.00)	1380.00 (877.50)	1189.00 (772.30)	1017.00 (673.40)	863.70 (582.30)
	Cord concentration	C_min_ (ng/mL)					61.49 (43.49)	69.33 (49.85)	71.62 (52.80)	70.08 (52.74)	66.45 (50.88)
		C_max_ (ng/mL)					77.97 (49.70)	88.84 (57.58)	91.86 (61.15)	89.38 (60.98)	84.16 (58.68)
EM CYP2D6	Recommended daily dose (mg)		175, 200	175, 200, 225	175, 200, 225, 250	200, 225, 250, 275	225, 250, 275	250, 275	275, 300	300	300
	CL (L/h)		66.97 (41.10)	74.37 (43.17)	72.33 (39.38)	81.45 (45.32)	91.63 (72.31)	106.60 (86.12)	123.40 (101.50)	149.60 (124.70)	175.90 (148.80)
	AUC (ng/mL·h)		1743.00 (994.60)	1751.00 (905.60)	1724.00 (922.30)	1749.00 (944.70)	1776.00 (1029.00)	1664.00 (960.70)	1595.00 (933.00)	1453.00 (855.50)	1251.00 (744.90)
	Cord concentration	C_min_ (ng/mL)					69.28 (46.30)	84.98 (56.66)	97.57 (65.93)	101.50 (68.97)	97.40 (66.77)
		C_max_ (ng/mL)					83.83 (51.01)	103.40 (62.61)	118.80 (72.98)	123.10 (76.28)	117.50 (73.72)
PM CYP2D6	Recommended daily dose (mg)		75, 100	75, 100	75, 100	100,	100	100	100	100, 125	100, 125
	CL (L/h)		31.44 (14.72)	31.77 (15.88)	32.48 (16.22)	33.39 (16.68)	35.11 (16.04)	36.57 (16.46)	38.12 (17.11)	39.97 (17.85)	42.18 (18.78)
	AUC (ng/mL·h)		3373.00 (1723.00)	3331.00 (1564.00)	3257.00 (1527.00)	3618.00 (1598.00)	3422.00 (1545.00)	3251.00 (1367.00)	3116.00 (1308.00)	3340.00 (1452.00)	3162.00 (1372.00)
	Cord concentration	C_min_ (ng/mL)					65.60 (33.83)	81.33 (39.24)	93.67 (45.16)	115.00 (56.94)	121.50 (59.97)
		C_max_ (ng/mL)					86.10 (38.67)	106.80 (44.91)	122.40 (51.55)	149.00 (65.13)	155.90 (68.07)

Mean (SD); C_min_, trough concentration; C_max_, maximum concentration; CL, clearance; AUC, Area-under-the-curve; ↓, initiation of foetoplacental PBPK model.

## Data Availability

The data presented in this study are available in the article and supplementary material.

## Supplementary Materials

The following supporting information can be downloaded at: https://www.mdpi.com/article/10.3390/metabo12121281/s1, Figure S1: Fetoplacental compartment permeability-limited model; Figure S2: Percentage of Ultrarapid Metaboliser (UM) population with trough concentration below 60 ng/mL; Figure S3: Percentage of UM population with trough concentration above 230 ng/mL; Figure S4: Percentage of Extensive Metaboliser (EM) population with trough concentration below 60 ng/mL; Figure S5: Percentage of EM population with trough concentration above 230 ng/mL; Figure S6: Percentage of Poor Metaboliser (PM) population with trough concentration below 60 ng/mL; Figure S7; Percentage of PM population with trough concentration above 230 ng/mL.
