# Supplementary material for: Optimising Fluvoxamine Maternal/Fetal Exposure during Gestation: A Pharmacokinetic Virtual Clinical Trials Study
Source: Metabolites. 2022 Dec 16;12(12):1281. doi: 10.3390/metabo12121281 (PMC9782298; doi:10.3390/metabo12121281)
Supplement: Supplementary file 1 [file metabolites-12-01281-s001.zip › metabolites-2094176-supplementary.pdf]

Optimising Fluvoxamine Maternal/Fetal Exposure during Gestation: A Pharmacokinetic Virtual Clinical Trials Study

Khairulanwar Burhanuddin and Raj Badhan *

School of Pharmacy, College of Health and Life Science, Aston University, Birmingham B4 7ET, UK.
* Correspondence: r.k.s.badhan@aston.ac.uk

Supplementary materials

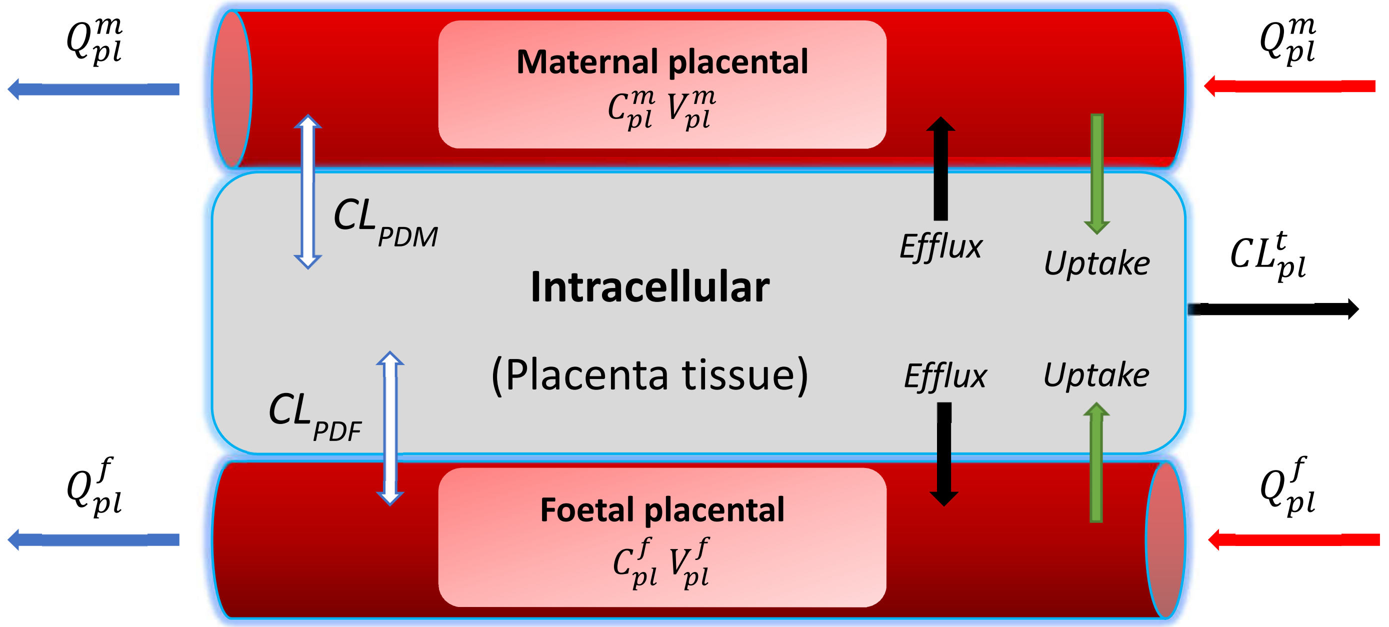

Figure S1. Fetoplacental compartment permeability-limited model.

CL_{PDM}, maternal–placental permeability clearance; CL_{PDF}, placental–foetal permeability clearance; CL_{pl}^{t}, tissue–placenta permeability clearance; Q_{pl}^{m}, maternal blood flow rate; Q_{pl}^{f}, foetal blood flow rate; C_{pl}^{m}, maternal–placental concentration; C_{pl}^{f}, foetal–placental concentration; V_{pl}^{m}, maternal–placental volume; V_{pl}^{f}, foetal–placental volume.

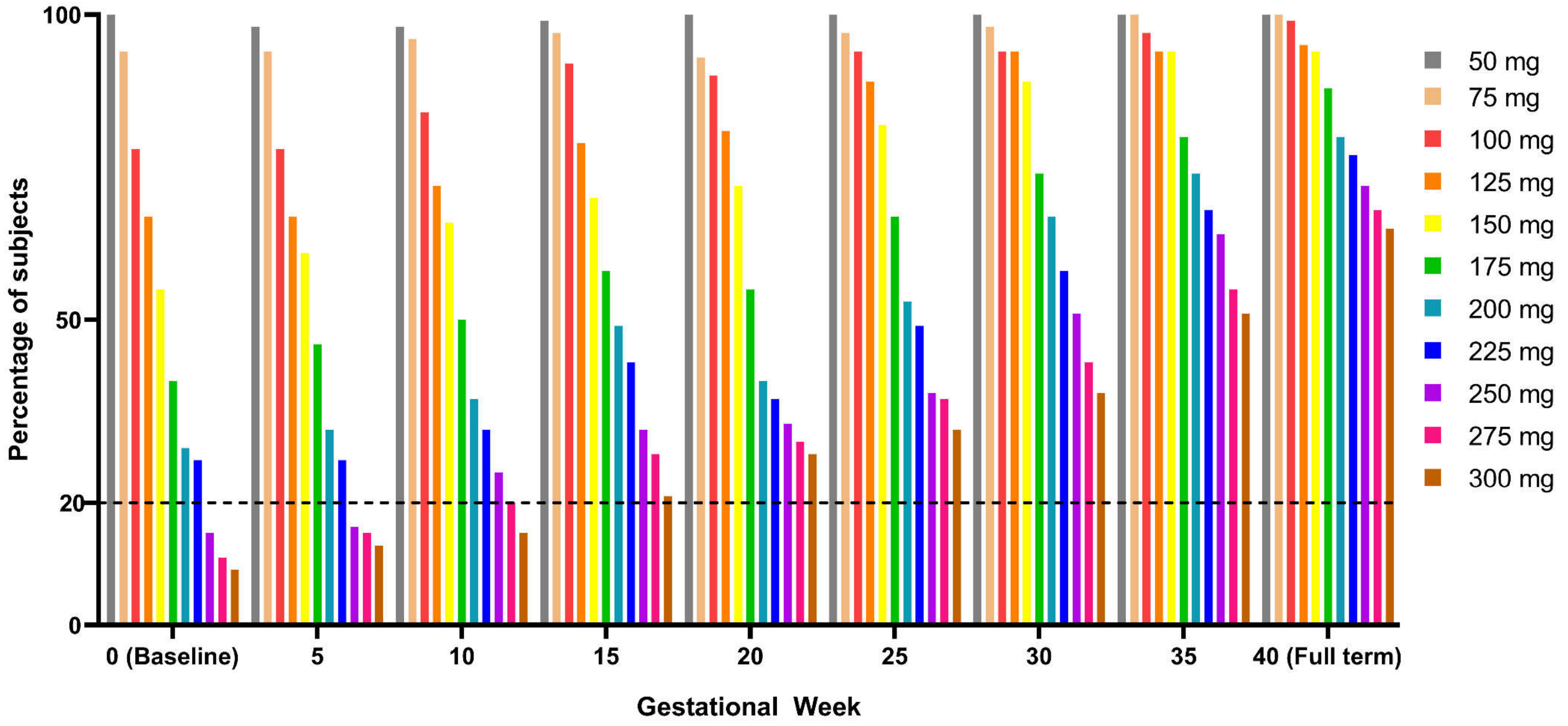

Figure S2. Percentage of Ultrarapid Metaboliser (UM) population with trough concentration below 60 ng/mL. The dashed horizontal line represents the threshold of 20% of subjects.

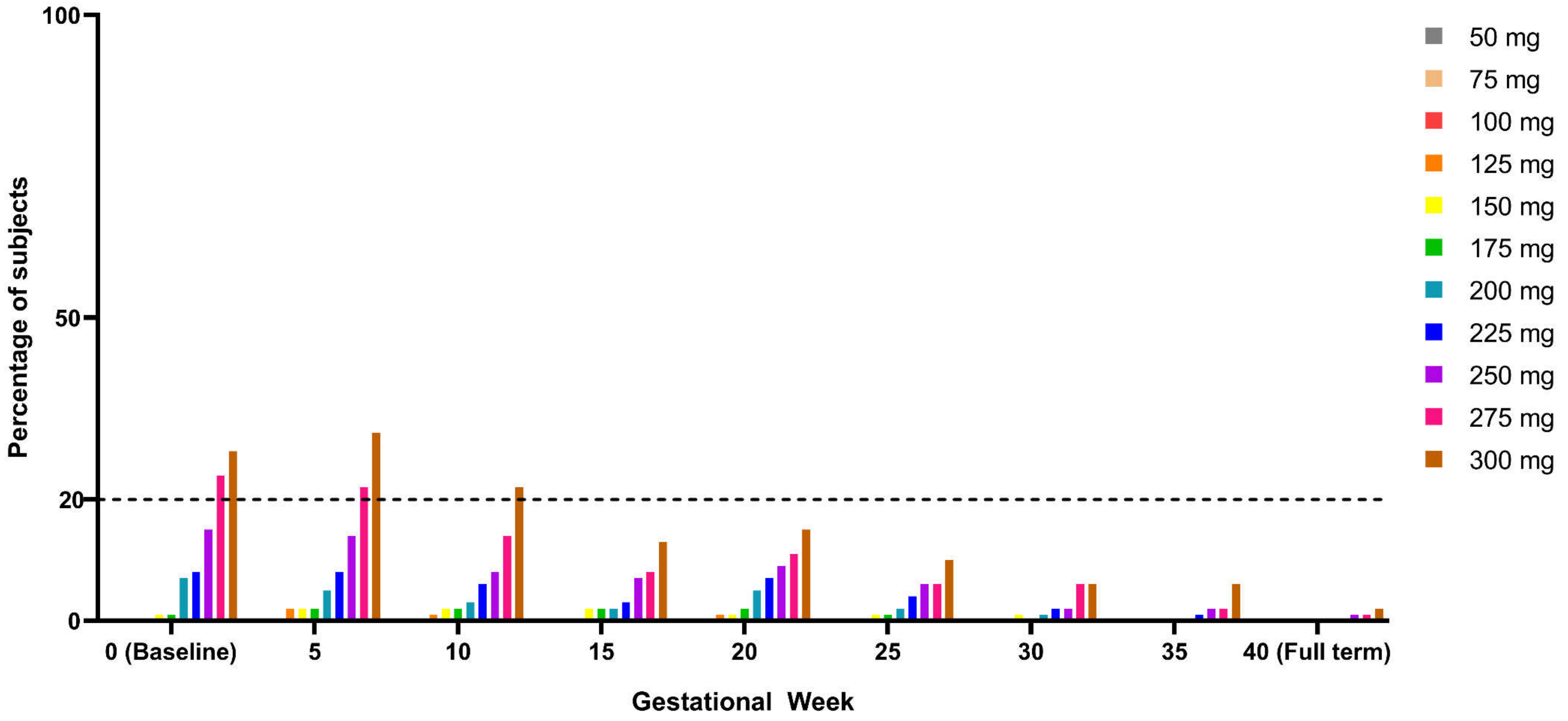

Figure S3. Percentage of UM population with peak concentration above 230 ng/mL.
The dashed horizontal line represents the threshold of 20% of subjects.

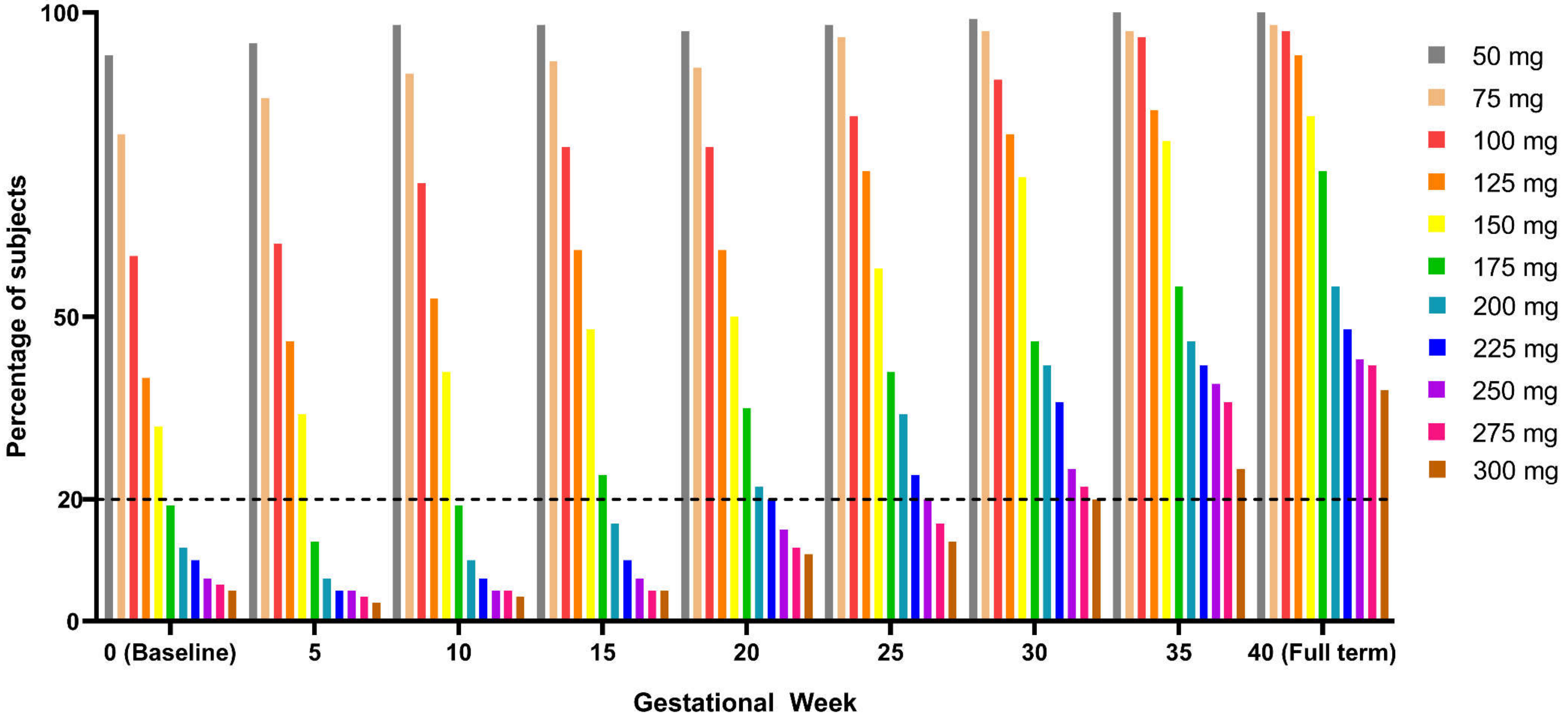

Figure S4. Percentage of Extensive Metaboliser (EM) population with trough concentration below 60 ng/mL. The dashed horizontal line represents the threshold of 20% of subjects.

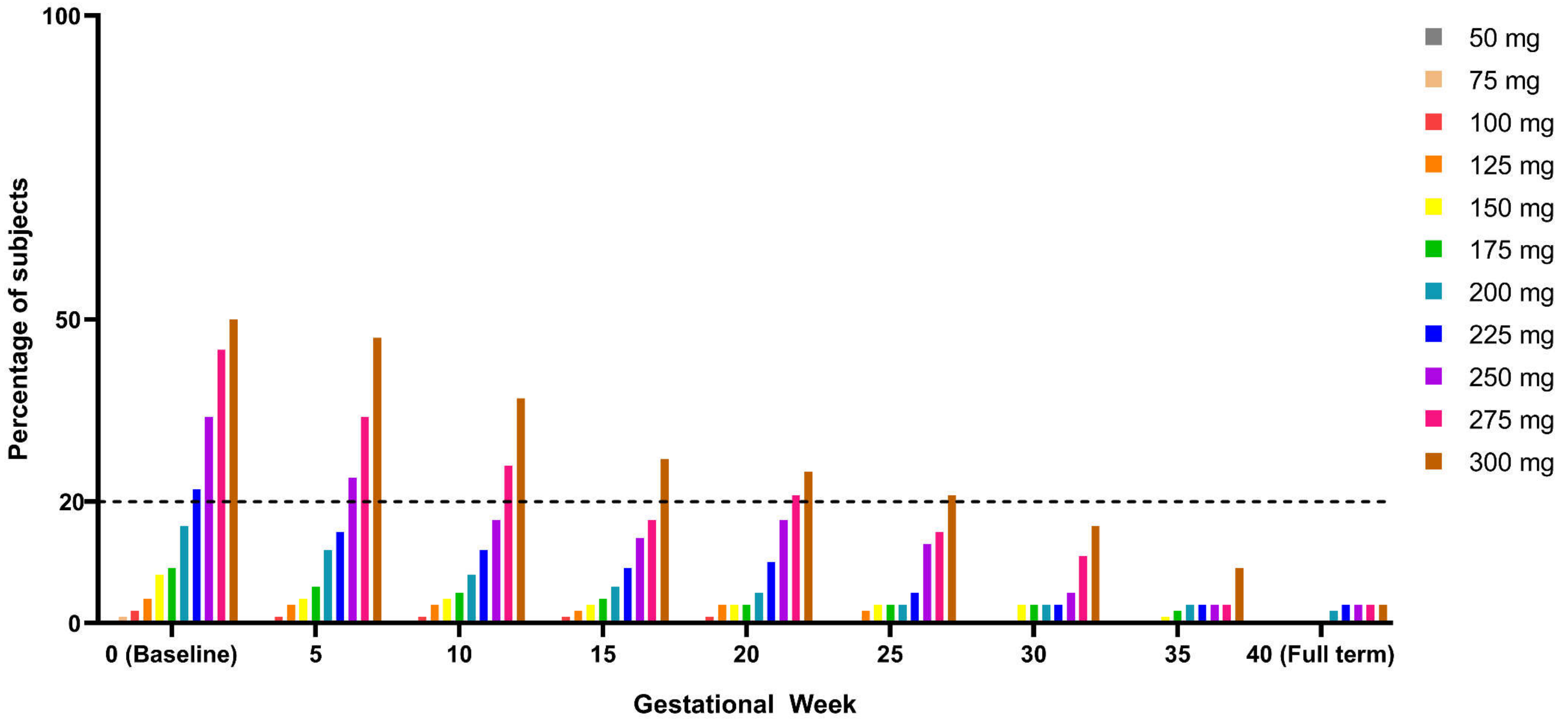

Figure S5. Percentage of EM population with peak concentration above 230 ng/mL.
The dashed horizontal line represents the threshold of 20% of subjects.

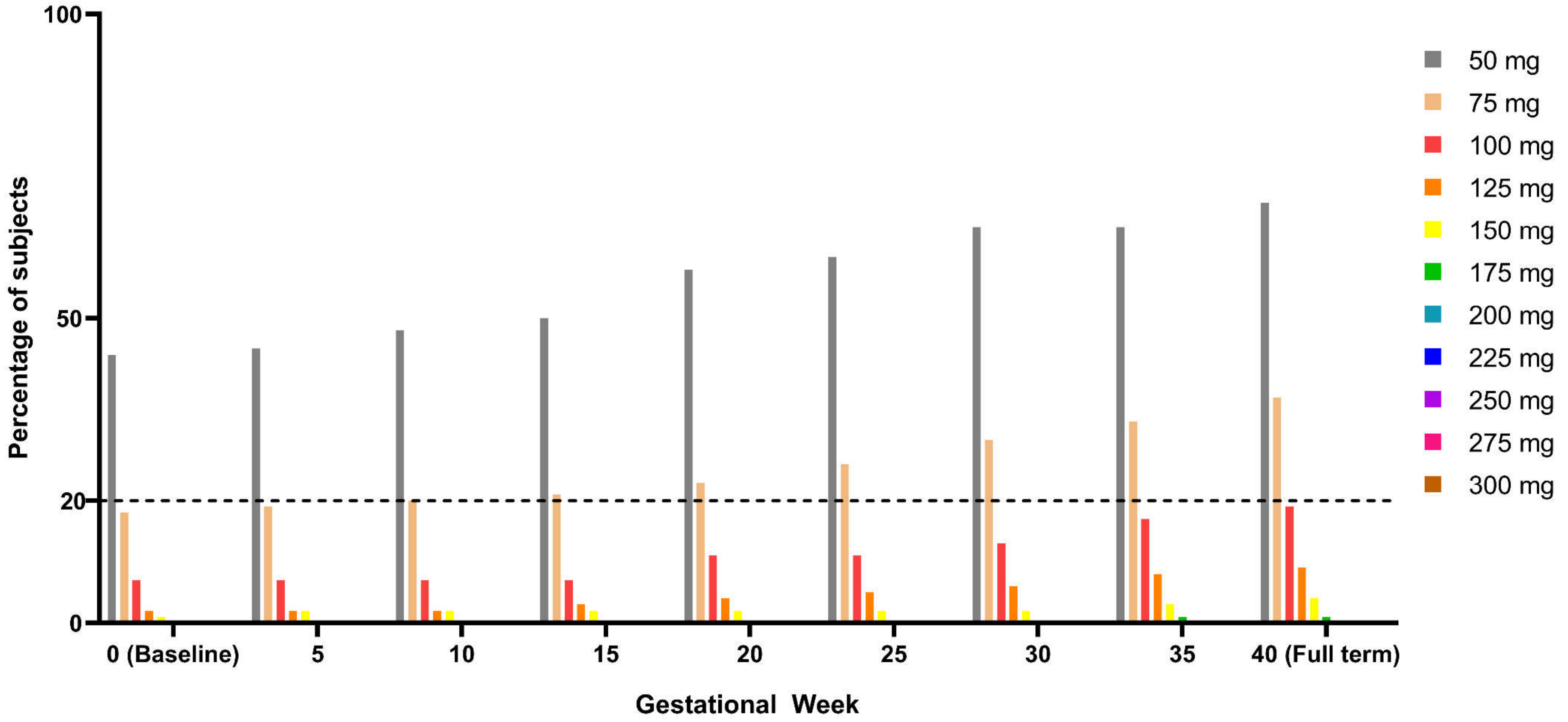

Figure S6. Percentage of Poor Metaboliser (PM) population with trough concentration below 60 ng/mL. The dashed horizontal line represents the threshold of 20% of subjects.

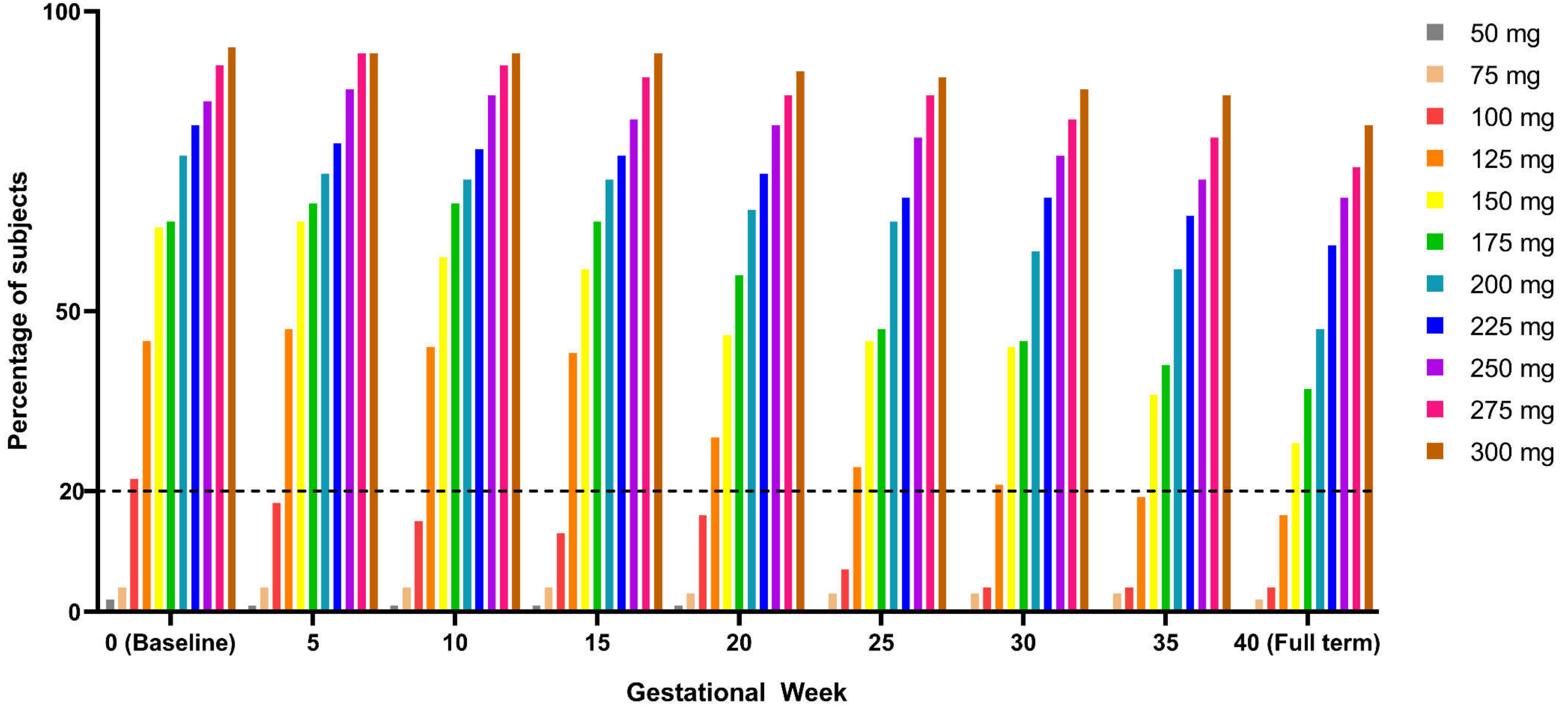

Figure S7. Percentage of PM population with peak concentration above 230 ng/mL. The dashed horizontal line represents the threshold of 20% of subjects.
